# Phosphate-Starvation-Inducible S-Like RNase Genes in Rice Are Involved in Phosphate Source Recycling by RNA Decay

**DOI:** 10.3389/fpls.2020.585561

**Published:** 2020-11-30

**Authors:** Yun-Shil Gho, Heebak Choi, Sunok Moon, Min Yeong Song, Ha Eun Park, Doh-Hoon Kim, Sun-Hwa Ha, Ki-Hong Jung

**Affiliations:** ^1^Graduate School of Biotechnology, Kyung Hee University, Yongin, South Korea; ^2^Department of Life Science, College of Life Science and Natural Resources, Dong-A University, Busan, South Korea

**Keywords:** rice, S-like RNases, phosphate starvation, phosphate recycling, RNA degradation

## Abstract

The fine-tuning of inorganic phosphate (Pi) for enhanced use efficiency has long been a challenging subject in agriculture, particularly in regard to rice as a major crop plant. Among ribonucleases (RNases), the RNase T2 family is broadly distributed across kingdoms, but little has been known on its substrate specificity compared to RNase A and RNase T1 families. Class I and class II of the RNase T2 family are defined as the S-like RNase (RNS) family and have showed the connection to Pi recycling in Arabidopsis. In this study, we first carried out a phylogenetic analysis of eight rice and five Arabidopsis *RNS* genes and identified mono-specific class I and dicot-specific class I RNS genes, suggesting the possibility of functional diversity between class I RNS family members in monocot and dicot species through evolution. We then compared the *in silico* expression patterns of all *RNS* genes in rice and Arabidopsis under normal and Pi-deficient conditions and further confirmed the expression patterns of rice *RNS* genes via qRT-PCR analysis. Subsequently, we found that most of the *OsRNS* genes were differentially regulated under Pi-deficient treatment. Association of Pi recycling by RNase activity in rice was confirmed by measuring total RNA concentration and ribonuclease activity of shoot and root samples under Pi-sufficient or Pi-deficient treatment during 21 days. The total RNA concentrations were decreased by < 60% in shoots and < 80% in roots under Pi starvation, respectively, while ribonuclease activity increased correspondingly. We further elucidate the signaling pathway of Pi starvation through upregulation of the *OsRNS* genes. The 2-kb promoter region of all *OsRNS* genes with inducible expression patterns under Pi deficiency contains a high frequency of P1BS cis-acting regulatory element (CRE) known as the OsPHR2 binding site, suggesting that the OsRNS family is likely to be controlled by OsPHR2. Finally, the dynamic transcriptional regulation of *OsRNS* genes by overexpression of *OsPHR2*, *ospho2* mutant, and overexpression of *OsPT1* lines involved in Pi signaling pathway suggests the molecular basis of *OsRNS* family in Pi recycling via RNA decay under Pi starvation.

## Introduction

Inorganic phosphate (Pi) is a crucial component of major organic molecules such as RNA, DNA, and ATP in all organisms. In plants, Pi (H_2_PO_4_^–^ or HPO_4_^–2^) is mainly absorbed by plant roots in the soil. Pi starvation promotes changes in root system architecture and reduces crop productivity. To overcome Pi starvation in plants, plants enhance the activity of RNases, phosphatases, and Pi transporters that promote Pi acquisition by roots (Vance et al., 2003; Plaxton and Tran, 2011; Zhu et al., 2019). Ribonucleases are ubiquitous components of cells that catalyze the cleavage of RNA and act on either single-stranded, double-stranded, or DNA–RNA hybrid substrates. Ribonucleases, which hydrolyze RNA to 3′ mononucleotides via 2′,3′ cyclic nucleotides, have been classified into three functional groups: the RNase A, RNase T1, and RNase T2 families. The RNase A family consists of proteins with a molecular mass between 13 and 14 kDa and either an alkaline (7–8) or weakly acidic (6.5–7) pH preference; moreover, they constitute a group of homologous proteins that have been isolated from many vertebrates, but not from invertebrates (Beintema et al., 1997; Beintema and Kleineidam, 1998). In turn, proteins in the RNase T1 family have a molecular mass of ∼12 kDa and pH optima between 7 and 8, and are found in fungi and bacteria. Finally, the RNase T2 family includes RNases with an average molecular mass around 25 kDa that were originally categorized as having acid RNase activity (Irie, 1999; Deshpande and Shankar, 2002). In addition, RNase T2 enzymes are transferase-type endoribonucleases that produce oligonucleotides and/or mononucleotides with a terminal 3′ phosphate via a 2′,3′ cyclic phosphate intermediate. The RNase T2 family exists in virtually all eukaryotes and seems to play important roles in a variety of biological processes.

The classification of the RNase T2 family in plants into three classes (I–III) is based on phylogenetic analyses (Igic and Kohn, 2001; MacIntosh et al., 2010; Ramanauskas and Igic, 2017). Some of plant class III RNases are involved in self-incompatibility, but in the class I and II RNases, none of the genes have been involved in self-incompatibility. Plant class I RNases are involved in abiotic stress responses such as salt stress and phosphate starvation stresses (Bariola et al., 1999; MacIntosh et al., 2010; Zheng et al., 2014); plant class II RNases participate in phosphate starvation (Bariola et al., 1999) and senescence (Taylor et al., 1993), and also has a housekeeping role in recycling ribosomal RNA (rRNA) (Hillwig et al., 2011; MacIntosh and Bassham, 2011; Floyd et al., 2015, 2017; Morriss et al., 2017); and besides self-incompatibility, some class III RNases are involved in stress such as phosphate starvation without being involved in self-incompatibility (Rojas et al., 2013, 2015, 2018). Thus, we defined plant class III RNases as S-RNases, and plant class I and II RNases as S-like RNases.

Several RNase genes have been identified in dicots, including *Arabidopsis*, *Nicotiana benthamiana* (tobacco), and *Solanum lycopersicum* (tomato) (Taylor et al., 1993; Bariola et al., 1994; Hillwig et al., 2011). Recent studies reported an association between class I RNSs and senescence and environmental signals (Bariola et al., 1999; MacIntosh et al., 2010; MacIntosh and Bassham, 2011), including inorganic phosphate (Pi) starvation, in *Arabidopsis* and *Nicotiana* (Rojas et al., 2013); in contrast, the class II RNS genes are constitutively expressed and likely perform housekeeping functions.

In cultivated *S. lycopersicum* (tomato) cells, the *RNase LE* and *RNase LX* genes are induced under Pi starvation (Kaletta et al., 1998). Two *Arabidopsis* RNase T2 genes, *RNS1* and *RNS2*, are also expressed under Pi starvation (Taylor et al., 1993; Bariola et al., 1994, 1999). *RNS2* and *RNase LX* are upregulated during senescence (Taylor et al., 1993; Lers et al., 1998). Although several studies reported the genome-wide identification of S-like RNase family genes in dicot plants, the global understanding of this family remains limited in monocot plants, including rice. Transcriptome analysis is a very simple and powerful tool that can be used to obtain functional clues for genes with uncharacterized features. Several studies have revealed important components of the sensing and signaling networks involved in Pi starvation responses in rice. *OsPHR2* (*Rice Phosphate Starvation Response 2*), which is an *AtPHR1*-like gene, has been reported as one of the central regulators of Pi signaling pathway because it regulates the expression of *Osa-miR399* and *OsPHT1s* by binding to the P1BS elements in the promoters of Pi-starvation-induced (PSI) genes (Zhou et al., 2008; Liu et al., 2010; Li et al., 2015). P1BS is also present in the promoters of many key PSI genes (Li et al., 2015; Ruan et al., 2015; Gho et al., 2018). *Osa-miR399* represses the expression of *OsPHO2*, a ubiquitin-conjugating E2 enzyme, which triggers the degradation of several *OsPHT1* family genes under normal conditions (Liu et al., 2010; Wu and Wang, 2011; Sun et al., 2012; Cao et al., 2014). Thus, OsPHR2, miR399, OsPHO2, and OsPTs are significant components of the Pi-signaling network. In this study, we carried out a comparative phylogenetic analysis using five *Arabidopsis* and eight rice *RNS* genes. Based on a sequence-similarity analysis and domain organization, we assigned two subgroups to this family. We also found that the class I *RNS* genes in rice, unlike those in *Arabidopsis*, a representative dicotyledonous plant, were spatially regulated in response to Pi starvation and various environmental stresses. In addition, the amount of total RNA was decreased by 60% in shoots and 80% in roots, respectively, under Pi starvation; concomitantly, RNase activity was enhanced under Pi starvation. Finally, a detailed data analysis of *RNS* family genes in rice associated with phosphate use efficiency is presented and the functional significance and further applications of these findings are discussed.

## Materials and Methods

### Plant Materials

Rice (*Oryza sativa* L. cv. *Dongjin*) seeds were germinated on Murashige Skoog medium under controlled conditions of 28°C day/25°C night temperatures, 8-h light/16-h dark cycle, and 78% relative humidity after sterilization with 50% (w/v) commercial bleach for 30 min with gentle shaking. For anatomical expression analysis, roots, leaf sheaths, leaf blades, panicles before heading, flowers at the heading stage, and seeds at 10 and 15 days after pollination were harvested to extract total RNAs. Four biological replicates were prepared and analyzed independently. For differential Pi-starvation-inducible expression analysis in shoots and roots, we used rice cv. *Dongjin* seedlings grown for 21 days in Pi-sufficient (0.320 mM Pi) or Pi-deficient (0 mM Pi) Yoshida solution (Cock et al., 1976) after germination for 10 days in MS media. The pH of the culture solution was adjusted to pH 5.5 using 6 M NaOH every 1 day, and total Yoshida solution was replaced every 3 days. In all hydroponic experiments, seedlings were directly grown in each of the culture solutions (8 L) with an 8-h light (28°C)/16-h dark (22°C) photoperiod.

### RNA Extraction, RNA Concentration, Semi-Quantitative RT–PCR, and Real-Time PCR

The anatomical tissues, phosphate stress-treated samples, and roots and shoots of *OsPHR2*-OX, *OsPT1*-OX, and *ospho2* were frozen in liquid nitrogen and ground with a Tissue Lyzer II (Qiagen; Hilden, Germany). RNAs were extracted with the RNAiso Plus Kit according to the manufacturer’s protocol (Takara Bio; Kyoto, Japan). Complementary DNA (cDNA) was synthesized. To compare RNA concentration in roots and shoots between phosphate-deficient plants and phosphate-sufficient plants, we used 40 mg for the shoot samples and 60 mg for the root samples to isolate RNA using the RNAiso Plus Kit and melted them into 50 μl of distilled water (DW). This experiment was repeated three times using the same amount of independent samples and was measured by UV spectroscopy (Thermo Scientific NanoDrop 2000). To determine the tissue-preferential expression patterns, phosphate-deficient expression, and the expression patterns in the above three genetic resources via real-time PCR, we used *OsUbi5* as the reference gene (Jain et al., 2006). We used cycling conditions of 95°C for 15 s, 57°C for 30 s, and 72°C for 60 s. This experiment was repeated three times by using independent biological replicates. Relative transcript levels and fold changes were calculated by the 2^Δ*Ct*^ and 2^ΔΔ*Ct*^ methods (Schmittgen and Livak, 2008), respectively.

### Preparation of Phosphate Signaling Mutants

To confirm the expression analysis of the *OsRNS* family gene regulated by the *PSI signaling gene*, we used *rice phosphate 2* (*ospho2*) T3 mutants, *rice phosphate transporter1* overexpressing T5 line (*OsPT1*-OX), and *rice phosphate* starvation *response 2* overexpressing T2 line (*OsPHR2*-OX). The *ospho2* T-DNA insertion mutant lines, PFG_1C-06032, were obtained from RiceGE^1^. The *OsPT1*-OX line, which expresses high-affinity phosphate transporters in rice, was obtained from Dong A University, South Korea (Seo et al., 2008). The overexpression of *OsPHR2* was generated. The 1326 bp of the coding region of *OsPHR2* including the Kozak sequence was amplified from the rice (*O. sativa* L. cv. *Dongjin*) cDNA using the primers listed in Supplementary Table S1. The PCR products were directly cloned into the pGA3438 vector by Infusion Cloning (Infusion HD Cloning Kit, Clontech, 639644, California, United States) (Kim et al., 2009). The expression vectors were transferred to *Agrobacterium tumefaciens* strain LBA4404 by transformation and co-cultivation in rice callus (*O. sativa* L. cv. *Dongjin*), as described previously (Moon et al., 2019). Three mutants used in this study were further confirmed by qRT-PCR with *rice ubiquitin 5* (*OsUbi5*, *LOC_Os01g22490*) as the reference gene. The primers used in these analyses are summarized in Supplementary Table S1.

### Multiple Sequence Alignment and Phylogenetic Analysis

To perform a phylogenetic tree analysis of RNS family genes in two monocot plants (rice and *Zea mays*) and four dicot plants (Arabidopsis, *Brassica rapa*, *S. lycopersicum*, and *Medicago truncatula*), we collected RNS family protein sequences from six plant species using the Interactive Phylogenetics Module tool based on rice RNS locus ID from the PLAZA 4.0 database^2^ (Van Bel et al., 2018). The multiple alignment of the amino acid sequences was carried out using the ClustalX program version 2.0.11 (Higgins et al., 1996). Phylogenetic analysis was performed using MEGA 7 under neighbor-joining tree method (Tamura et al., 2011). In addition, to perform a phylogenomic analysis of *RNS* genes in rice and *Arabidopsis thaliana*, we collected eight family members from the Rice Genome Annotation Project using locus IDs (RGAP^3^) and five *Arabidopsis* family members from a previous report (MacIntosh et al., 2010). The multiple alignment of the amino acid sequences was carried out using the ClustalX program version 2.0.11 (Higgins et al., 1996). Phylogenetic analysis was performed using MEGA 6 and the following parameters: neighbor-joining tree method, complete deletion, and bootstrap with 1000 replicates (Tamura et al., 2011).

### Meta-Analysis of Tissue Expression Profiles

The integration of transcriptomes into a phylogenetic context can direct experimental strategies for further functional analysis (Jung et al., 2010). Therefore, we used meta-analysis of the expression profiles of *RNS* genes in six tissues/organs based on data from 983 Affymetrix arrays downloaded from the NCBI gene expression omnibus (GEO^4^) (Cao et al., 2012). We then uploaded the log_2_ normalized intensity data in tab-delimited text format into Multiple Experiment Viewer (MEV^5^) and illustrated these data using heat maps. In addition, we analyzed the meta-expression patterns of *RNS* genes in six tissues/organs of *Arabidopsis* using the *Arabidopsis* Affymetrix microarray data series GSE5630, GSE5633, GSE5631, GSE5632, GSE5634, GSM943445, and GSM943446. Similar to the rice data analysis, we generated meta-expression data. For comparative gene expression analysis, we used data from six tissue/organ types for both rice and *Arabidopsis*. As a result, the meta-expression data of all *RNS* genes were analyzed. The log_2_ intensity ranges of microarray data were differently shown between rice and *Arabidopsis* by a range from 5 to 15 and a range from 5 to 13, respectively, due to normalization. Yellow-colored boxes indicate a high level of expression and blue ones denote a low level of expression. Integrated meta-expression data were used to determine functional conservancy in terms of anatomy between rice and *Arabidopsis RNS* ortholog pairs.

### Global Identification of Rice *RNS* Genes Stimulated Under Phosphate Starvation Using Public RNA-Sequencing Data

To identify the role of *RNS* genes under phosphate starvation, we used the RNA-seq data from rice and microarray data from *Arabidopsis* obtained under phosphate-starvation conditions in a recent report (Woo et al., 2012; Secco et al., 2013; Gho et al., 2018). The fragments per kilobase of transcript per million mapped reads (FPKM) values from whole samples used in this study were downloadable from the National Center for Biotechnology Information^6^ under accession number SRA097415. *Arabidopsis* microarray data (GSE34004) were downloaded from the NCBI GEO^4^. Subsequently, we selected the generation of fold changes between the phosphate-sufficient condition and phosphate starvation for 21 days + 1 h (21 d + 1 h) in shoots and roots in rice and for 10 days in shoots and roots of *Arabidopsis*. Moreover, we confirmed additional fold changes to compare Pi recovery conditions (+1 h) after phosphate starvation for 21 days with phosphate starvation in rice and Pi recovery conditions for 3 days after phosphate starvation for 10 days with phosphate starvation in *Arabidopsis* (Supplementary Figure S2). We used average normalized three FPKM values and three intensity values of *RNS* genes from RNA-seq and microarray data. Heat map images were produced using the MEV software (Chu et al., 2008).

### RNase Activity

RNase activity was analyzed by agarose gel electrophoresis. Total RNA was prepared from 21-day-old rice shoot tissues using the purelink plant RNA reagent kit (Invitrogen). Crude protein extracts were prepared from 31-day-old rice plants under diverse experimental conditions (10 days of solid MSO followed by 21 days of P-sufficient or -starvation condition in a hydroponic culture system with shoot and root tissues). After each sample was ground with liquid nitrogen, the samples were vigorously mixed with 10 mM Tris–HCl buffer (pH 7.4) in 4°C. The mixed samples were centrifuged for 10 min, at 12,000 r/min, and 4°C, and the supernatant was calibrated with Quanti-iT Protein Assay (Thermo scientific). For each lane, 1 μg of total RNA was incubated in 25 mM Tris–HCl buffer (pH 7.4) containing 25 mM KCl and 5 mM MgCl_2_ in the presence of 0.5 and 0.1 μg of total protein from different samples at 30°C for 5 min. After incubation, the RNA samples were loaded onto 1.2% agarose gels containing 1 × MOPS and 2.2 M formaldehyde. This experiment was repeated three times using independent biological replicates.

### Assessment of the Integrity of RNA Samples Using Electropherograms

An integrity assessment analysis of RNA samples was performed by a company (Macrogen, Inc., South Korea) using an Agilent 2100 Bioanalyzer and RNA Bioanalyzer Pico 6000 chip (Agilent Technologies, Inc., Santa Clara, CA, United States).

### Measurements of Total Pi Content in Plants

Samples were ground in liquid nitrogen after fresh-weight measurement (about 50–70 mg) and then homogenized in 200 μl of 10% perchloric acid (PCA). After adding 1.8 ml of 5% PCA, the homogenate was mixed and placed on ice for 30 min. After centrifugation at 10,000 × *g* for 10 min at 4°C, the supernatant was used to measure Pi using the molybdate-blue method: 0.4% ammonium molybdate melted in 0.5 M H_2_SO_4_ (solution A) was mixed with 10% ascorbic acid (solution B) (A:B = 6:1). Two milliliters of this solution was added to 1 ml of the sample solution and incubated in a water bath at 40°C for 20 min. The absorbance was measured at 820 nm after cooling on ice for 5 min, and the Pi content was calculated from the absorbance value per fresh weight. This experiment was repeated three times by using independent biological replicates. We conducted inorganic Pi measurement according to Yang et al. (2014).

## Results

### Identification of Rice S-Like RNase Family (*RNS*) Genes and Comparative Phylogenetic Analysis With *Arabidopsis RNS* Genes

**FIGURE 1:**
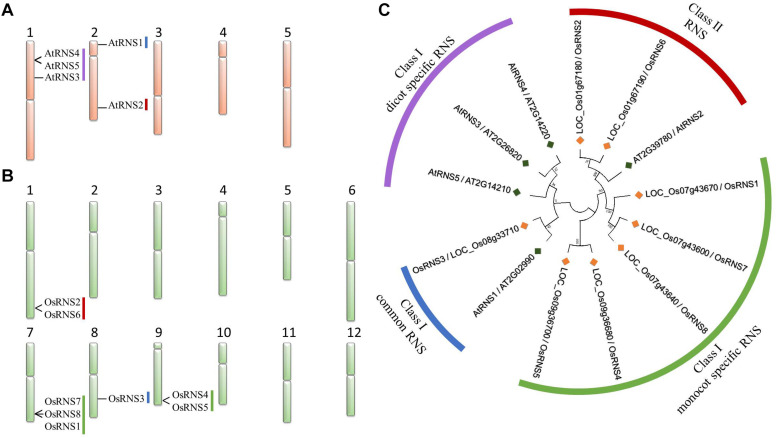
Chromosome map and phylogenetic tree of rice and *Arabidopsis RNS* family genes. The chromosome map of *Arabidopsis RNS* family genes **(A)** and *rice RNS* family genes **(B)**. Phylogenetic tree of the RNS family comprising eight rice (*Oryza sativa*) and five *Arabidopsis* RNS proteins **(C)**. The phylogenetic tree was built using the neighbor-joining method with a bootstrap value of 1000 using MEGA 7.

MacIntosh et al. (2010) performed a phylogenetic analysis of the *RNS* family that included eight rice and five *Arabidopsis* RNS genes present in Figures 1A,B. Among them, *OsRNS1*, *OsRNS3*, *OsRNS4*, *OsRNS5*, and *OsRNS8* are class I *RNS* genes, while *OsRNS2* and *OsRNS6* are class II *RNS* genes (MacIntosh et al., 2010). However, in contrast with *Arabidopsis*, the role of the individual family members was not well characterized in rice. To overcome this limitation, we attempted to assign biological functions to the *RNS* genes in rice according to tissues/organs or various stresses, including Pi starvation. Although, in the previous study, plant RNSs were already clustered based on phylogenetic analyses (MacIntosh et al., 2010; Ramanauskas and Igic, 2017), we have redrawn the phylogenetic tree using two monocot [rice (eight genes) and *Z. mays* (five genes)] and four dicot [*Arabidopsis* (five genes), *B. rapa* (eight genes), *S. lycopersicum* (six genes), and *M. truncatula* (seven genes)] RNSs to identify the evolutionary difference between class I and class II RNS in monocot and dicot plants. As a result, there was no significant difference in class II RNS family between rice and Arabidopsis as described in the previous report (MacIntosh et al., 2010), but in class I RNS family, it has been identified that it was divided into three groups (class I monocot-common, class I monocot-specific, and class I dicot-specific RNS subgroups) in a little more detail (Supplementary Figure S1). These results have already been mentioned. The diversification of class I RNS is due to a process characterized by differential maintenance and expansion in different lineages by gene classification, i.e., rapid gene replication and inactivation that occurs differentially between lineages (Zhang et al., 2000; MacIntosh et al., 2010).

Six rice and four *Arabidopsis RNS* genes were assigned to class I, and two rice and one *Arabidopsis RNS* gene were assigned to class II (Figure 1C). We found evolutionary conserved RNS genes between rice and *Arabidopsis*, such as *LOC_Os08g33710*/*OsRNS3*, which is an ortholog of *A. thaliana AT2G02990/AtRNS1* in class I *RNS* genes, whereas *LOC_Os01g67180/OsRNS2* and *LOC_Os01g67190/OsRNS6* are orthologs of *AT2G39780/AtRNS2* in class II *RNS* genes (Supplementary Table S2). We hypothesized that rice and *Arabidopsis RNS* genes that are clustered in the same subgroup have similar biological functions. Conversely, *AT1G26820/AtRNS3*, *AT1G14220/AtRNS4*, and *AT1G14210/AtRNS5* formed a class I dicot-specific RNS subgroup, whereas three rice *RNS* genes (*OsRNS1*, *OsRNS7*, and *OsRNS8*) on chromosome 7 and two rice *RNS* genes (*OsRNS4* and *OsRNS5*) on chromosome 9 formed a monocot-specific subgroup in class I *RNS* genes. In addition, three rice *RNS* genes on chromosome 7 and two genes on chromosome 9, together with *OsRNS2*, *OsRNS6*, *AtRNS4*, and *AtRNS5*, were tandemly duplicated and more likely functionally to be redundant. The estimation of the functional roles of *RNS* genes belonging to class I monocot-specific and dicot-specific RNS subgroups requires additional data, such as expression profiles and their functional studies.

### Functional Assignment of Rice and *Arabidopsis RNS* Genes Using a Meta-Expression Analysis in Six Tissues/Organs

**FIGURE 2:**
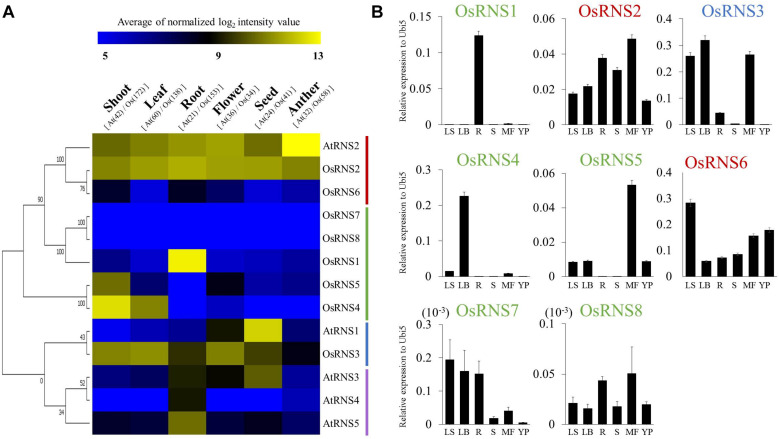
Anatomical meta-expression analysis of data from Affymetrix array platforms, and validation of the meta-expression patterns of eight *RNS* genes in various tissues/organs via qRT–PCR. **(A)** Affymetrix expression data from six tissues/organs were used to create the heat map with the MeV software. Blue, low levels of expression; yellow, high levels of expression. **(B)** qPCR was performed in six tissues/organs (LS, leaf sheath; LB, leaf blade; R, root; S, developing seeds; MF, mature flowers; and YP, young panicle). *X*-axis, tissues/organs used for qPCR analysis; *Y*-axis, relative expression level compared with that of *rice ubiquitin 5* (*OsUbi5, LOC_Os01g22490*). Four biological replicates were prepared and analyzed independently. The numbers on the heat map in **(A)** represent the number of Arabidopsis (At) and rice (Os) samples for each organ. In **(B)**, green letters indicate class I monocot-specific RNS; blue letters indicate class I common RNS; and red letters indicate class II RNS.

Although we inferred structural similarity among rice and *Arabidopsis RNS* genes clustered in the same subgroup using a phylogenetic analysis, additional data are required to determine the conservation of their biological function. Therefore, we performed a meta-expression analysis based on a large collection of microarray data for all *RNS* genes in six tissues/organs (Figure 2A). Among the *Arabidopsis* class I *RNS* genes, *AtRNS1* showed the highest expression in seeds and moderate expression in flowers, with *AtRNS3* exhibiting similar expression patterns. *AtRNS4* and *AtRNS5* showed root-preferred expression patterns, but the level of expression of *AtRNS5* was higher than that of *AtRNS4*. Among the rice class I *RNS* genes, *OsRNS4* and *OsRNS5* showed shoot-preferred expression patterns; moreover, *OsRNS5* exhibited moderate expression, while *OsRNS4* was expressed at a low level, in flowers. *OsRNS1* exhibited a root-preferred expression pattern, and *OsRNS3* was expressed at high level in shoots/leaves and flowers, and at moderate level in seeds, roots, and anthers. *OsRNS7* and *OsRNS8* exhibited a very low level of expression, and its expression might have been inhibited during evolution. *AtRNS4* and *AtRNS5* of *A. thaliana* and *OsRNS4* and *OsRNS5* of rice showed high amino acid similarity due to tandem redundancy and showed similar anatomical expression patterns, suggesting that they may have been cloned evolutionarily to enhance robustness of gene function. *OsRNS3* was clustered with *AtRNS1*, but the expression patterns of the two genes were dissimilar, suggesting functional divergence between them. The expression profiles of *RNS* genes belonging to monocot-specific and dicot-specific subgroups suggest that *OsRNS1* is a functional ortholog of *AtRNS4* and *AtRNS5*, because all of these genes had root-preferred expression patterns in the class I *RNS* family. In addition, the expression patterns of *OsRNS4* and *OsRNS5* informed that the class I *RNS* subgroup in rice plays unique roles in leaves or shoots compared with their *Arabidopsis* counterparts. Conversely, *AtRNS1* and *AtRNS3* might have a unique function in seed development compared with their counterparts in rice. In the class II *RNS* subgroup, *OsRNS2* might be a functional ortholog of *AtRNS2* because both genes showed ubiquitous expression patterns that were suggestive of a housekeeping role. In rice, this subgroup includes an additional member, *OsRNS6*, the expression pattern of which exhibited lower level of expression than both *OsRNS2* and *AtRNS2*. Based on the comparative anatomical expression analysis of *RNS* family genes between rice and *Arabidopsis*, we estimated that *AtRNS2* and *OsRNS2* have a conserved function as housekeeping genes in the class II *RNS* subgroup, whereas *OsRNS1*, *AtRNS4*, and *AtRNS5* have a conserved function in root development in the class I *RNS* subgroup.

### Validation of the Anatomical Meta-Expression Patterns of Rice *RNS* Genes Using qRT-PCR Analysis

To confirm the meta-anatomical expression patterns observed for the rice *RNS* genes, we carried out a qRT-PCR analysis using leaf sheaths, leaf blades, seedling roots, developing seeds, mature flowers, and young panicles. The three class I *RNS* genes (*OsRNS1*, *OsRNS4*, and *OsRNS5*) showed a tissue-dependent expression pattern: *OsRNS1* had a root-preferred expression pattern; *OsRNS4* was significantly expressed in leaf blades; and *OsRNS5* showed a mature flower-preferred expression pattern. Interestingly, *OsRNS3* was highly expressed in three above-ground tissues. *OsRNS2*, which was suggested to be a housekeeping gene, was ubiquitously expressed in all tested tissues/organs. *OsRNS6* also showed expression in all tested tissues, with the highest expression in leaf sheaths. The expression level of the remaining genes was very weak. In general, qRT-PCR data were well matched with the meta-expression data, indicating that meta-expression information based on a large collection of reference data is highly reliable and effective in estimating gene function (Figure 2B).

### A Close Association Between Rice *RNS* Genes and Phosphate Starvation Was Revealed by Both Meta-Expression and qRT–PCR Analyses

**FIGURE 3:**
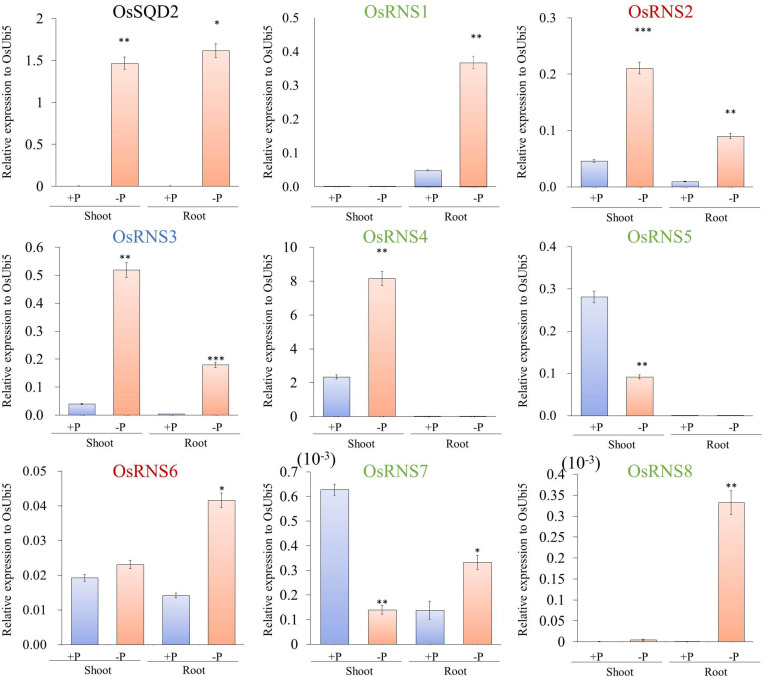
Expression analysis of eight *RNS* rice genes under phosphate starvation [+P (0.320 mM), –P (0 mM)] via qRT-PCR. *X*-axis, tissues and conditions used for the qPCR analysis; *Y*-axis, relative expression level compared with that of *OsUbi5*. Values are means ± SE (*n* = 4), and the asterisk indicates that the values of the phosphate starvation differ significantly (*P* < 0.05) compared with the phosphate-sufficient condition. ***P < 0.001, **P < 0.01, *P < 0.05, based on a *t*-test. Green letters indicate class I monocot-specific RNS; blue letter indicates class I common RNS; and red letters indicate class II RNS.

To determine the biological significance of rice *RNS* genes, we analyzed the diverse meta-expression data under various stress conditions. We found that rice *RNS* family genes showed significant differential expression under Pi starvation conditions (Woo et al., 2012; Secco et al., 2013; Gho et al., 2018). Supplementary Figure S1 shows the differential expression of these genes in the roots and shoots under Pi starvation compared with the normal condition. In class I RNS family, five rice *RNS* genes exhibited a > 2-fold upregulation in shoots or roots under Pi starvation. In contrast, *AtRNS1* alone showed a > 2-fold upregulation in shoots and roots under Pi starvation in class I RNS family. This result indicates that the rice *RNS* genes are more closely associated with Pi starvation than are *Arabidopsis RNS* genes. To confirm the expression patterns observed for the rice *RNS* genes under Pi starvation, we carried out a qRT-PCR analysis using shoots and roots that were incubated for 21 days under Pi starvation after germination for 10 days. To evaluate the quality of the samples under Pi starvation that were used for RT-PCR analysis, we first checked the expression patterns of two marker genes: rice *SULFOQUINOVOSYLDIACYLGLYCEROL 2* (Os*SQD2*, LOC_Os01g04920) and rice *PHOSPHATE TRANSPORTER 6* (*OsPT6*). We found that *OsSQD2* was significantly induced in both roots and shoots under Pi starvation, whereas *OsPT6* was only significantly induced in roots (Figure 3 and Supplementary Figure S3). Subsequently, we determined that five *RNS* genes (*OsRNS1*, *OsRNS3*, *OsRNS4*, *OsRNS7*, and *OsRNS8*) in the class I subgroup and two *RNS* genes (*OsRNS2* and *OsRNS6)* in the class II subgroup were significantly induced under Pi starvation. *OsRNS1* showed a root-preferred expression pattern, *OsRNS4* showed a shoot-preferred expression pattern, and *OsRNS3* was highly expressed in shoots and moderately expressed in roots. Although *OsRNS7* and *OsRNS8* were expressed at extremely low level, *OsRNS7* exhibited PSI repression in the shoot and *OsRNS8* exhibited Pi-starvation-inducible positive expression pattern in the root. Between *OsRNS2* and *OsRNS6*, which have been shown to be ubiquitously expressed in most tissues, *OsRNS2* was upregulated under Pi starvation in both roots and shoots, while *OsRNS6* was upregulated under Pi starvation only in roots (Figure 3). Conversely, *OsRNS5* was repressed in shoots under Pi starvation, as assessed in an RNA-seq analysis (Secco et al., 2013), with confirmation of its expression patterns in qRT-PCR analysis. Taken together, these results suggest that six (*OsRNS1*, *OsRNS2*, *OsRNS3*, *OsRNS4*, *OsRNS5*, and *OsRNS*6) out of eight RNS genes in rice are strongly associated with Pi starvation.

### Phosphate Deficiency Leads to RNA Degradation Mediated by *RNS* Genes

**FIGURE 4:**
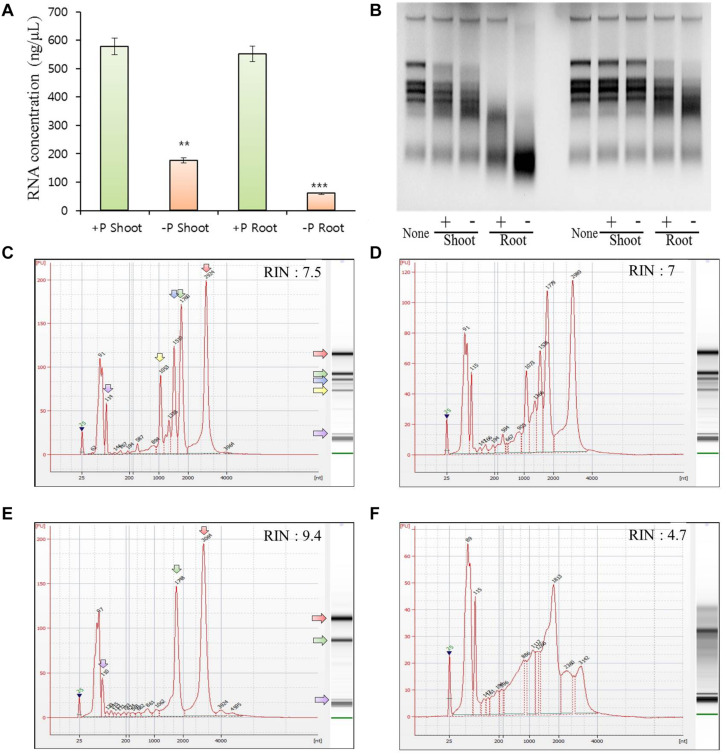
Tissue preferential RNA yield, RNase activity, and RNA integrity assessment of 21 days after germination rice grown under phosphate-sufficient (0.32 mM) or phosphate-starvation (0 mM) conditions. RNA yield of the samples from shoots and roots under phosphate-sufficient or -deficient conditions for 21 days using NanoDrop 2000 **(A)**. Four biological replicates were prepared and analyzed independently. Statistical significance was analyzed using Student’s *t*-test (^∗∗∗^*P* < 0.001; ^∗∗^*P* < 0.01). RNase activity was analyzed by agarose gel electrophoresis: 1 μg of total RNA from 21-day-old rice shoot tissues was incubated with 0.5 μg (left) and 0.1 μg (right) of protein from crude extracts from shoot and root samples grown in phosphate-sufficient or -deficient conditions for 21 days. The incubated total RNAs were equally measured (1 μg) and loaded **(B)**. Although we conducted experiments using three biological replicates, we presented only one dataset. Electropherograms of RNA samples from shoots **(C,D)** and roots **(E,F)** grown under phosphate-sufficient **(C,E)** or -deficient **(D,F)** conditions for 21 days. A standard size ladder in nucleotides (nt) is shown in the *X*-axis at the bottom of each graph. FU, fluorescent units at the *Y*-axis. The electrophoresis column generated by the program is located on the right side of each sample graph. The purple arrow indicates the peak of small RNAs. The positions of the 18S and 25S ribosomal RNAs are indicated by a green and red arrow, respectively. Compared with the root RNAs, the leaf RNAs exhibited additional fragments corresponding to the 23S and 16S chloroplastic rRNA fragments, respectively. The positions of the 16S and 23S ribosomal RNAs are indicated by a yellow and blue arrow, respectively.

To further test the relationship between Pi starvation and the function of *RNS* genes in rice, we measured the total RNA concentration using a spectrophotometer (Thermo Scientific NanoDrop 2000), ribonuclease activity by RNA gel electrophoresis assay, and the RNA integrity number (RIN) using a Bioanalyzer Pico 6000 chip (Agilent Technologies, Inc., Santa Clara, CA, United States) in samples from shoots and roots that were grown under phosphate-sufficient or -deficient conditions for 21 days. We found that the samples obtained from shoots and roots grown under Pi starvation exhibited a decrease of > 60–80% in the total RNA concentration compared with the normal condition, which was accompanied by increased ribonuclease activity in both shoots and roots under Pi starvation (Figures 4A,B).

Plant tissues have three types of rRNAs: chloroplastic (5S, 16S, and 23S rRNAs), cytosolic (5S, 18S, and 25S rRNAs), and mitochondrial (12S and 16S rRNAs). Compared with the root RNAs, the leaf RNAs exhibited additional fragments corresponding to the 23S and 16S chloroplastic rRNA fragments. Thus, they had lower RIN values than those of root samples (Babu and Gassmann, 2016). Our result also indicates that the RIN of shoot RNA was lower than that of root RNA (Figures 4C–F). RNAs from rice shoots grown under long-term Pi starvation exhibited a slightly lower RIN value compared with those obtained from plants grown under Pi-sufficient conditions; moreover, RNAs from rice roots grown under long-term Pi starvation exhibited markedly decreased RIN values compared with those obtained under Pi-sufficient conditions. We also found that the 25S rRNA was severely degraded after long-term Pi starvation in rice roots. Our results suggest that rice root RNAs are more sensitive to degradation in the presence of Pi starvation than are those from rice shoots (Figures 4C–F). Taken together, our findings indicate that the increase in ribonuclease activity triggered by the upregulation of *OsRNS* genes under Pi starvation might contribute to RNA degradation.

### Cis-Acting Regulatory Element (CRE) Analysis of *RNS* Family Gene Promoters via *in silico* Analysis

To identify consensus cis-acting regulatory elements (CREs) for PSI expression in eight *RNS* genes, we first extracted 2-kb sequences upstream of the ATG for these eight genes and analyzed the promoter sequences in the PLANTPAN 2.0 database. The *in silico* analysis of CREs revealed that 31 CREs were conserved among the eight genes; among them, we selected two consensus CREs in the promoters of the eight *RNS* genes as candidate CREs involved in Pi-starvation-inducible expression: P1BS/GNATATNC and WBOXNTERF3/TGACY (Supplementary Figure S4). The P1BS, which is an AtPHR1 binding site, is also present in the promoters of many crucial PSI genes (Li et al., 2015; Ruan et al., 2015; Gho et al., 2018). WBOXNTERF3 is one of the WRKY binding sequences, and previous studies have shown that *OsWRKY74*, *AtWRKY45*, and *AtWRKY75* are involved in regulating PSI responses in rice and *Arabidopsis* (Devaiah et al., 2007; Dai et al., 2016). It has also been reported that WBOXNTERF3 exists in the promoter area of high-affinity phosphate transporters (PHT1 group) in rice and wheat.

### Spatial Regulation of the *RNS* Family Genes in PSI Signaling

**FIGURE 5:**
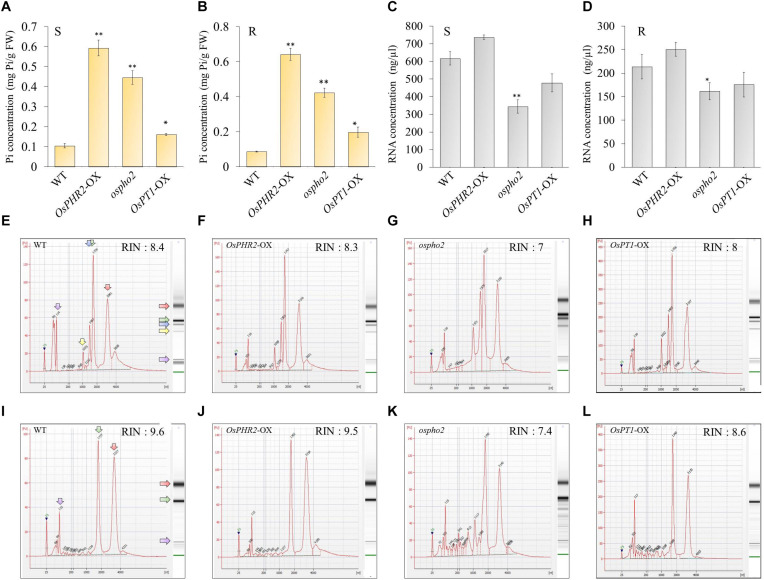
Pi content, RNA concentration, and RIN values of the bioanalyzer data from the roots and shoots of Phosphate Starvation Response 2 (*OsPHR2*-OX)-overexpressing, Phosphate 2 (*ospho2*) knockout, and phosphate transporter 1 (*OsPT1*-OX)-overexpressing lines in the phosphate-starvation-induced (PSI) pathway. Pi content of *OsPHR2*-OX, *ospho2*, and *OsPT1*-OX in the shoots **(A)** and roots **(B)** of 10-day seedlings. Determination of the RNA yield of *OsPHR2*-OX, *ospho2*, and *OsPT1*-OX in the shoots **(C)** and roots **(D)** of 10-day seedlings using NanoDrop 2000. Values are means ± SE (*n* = 3), and the asterisk indicates that the values of the mutants or Ox plants differ significantly (*P* < 0.05) compared with their corresponding wild types. Statistical significance was analyzed using Student’s *t*-test (^∗∗^*P* < 0.01; ^∗^*P* < 0.05). Electropherograms of RNA samples from WT **(E)**, *OsPHR2*-OX **(F)**, *ospho2* **(G)**, and *OsPT1*-OX **(H)** in shoots and WT **(I)**, *OsPHR2*-OX **(J)**, *ospho2* **(K)**, and *OsPT1*-OX **(L)** in roots. A standard size ladder in nucleotides (nt) is shown in the *X*-axis at the bottom of each graph. FU, fluorescent units at the *Y*-axis. The electrophoresis column generated by the program is located on the right side of each sample graph. The positions of the 18S and 25S ribosomal RNAs are indicated by a green and red arrow, respectively. The positions of the 16S and 23S ribosomal RNAs are indicated by a yellow and blue arrow, respectively. Although we conducted experiments using three biological replicates with similar results, we presented only one dataset.

**FIGURE 6:**
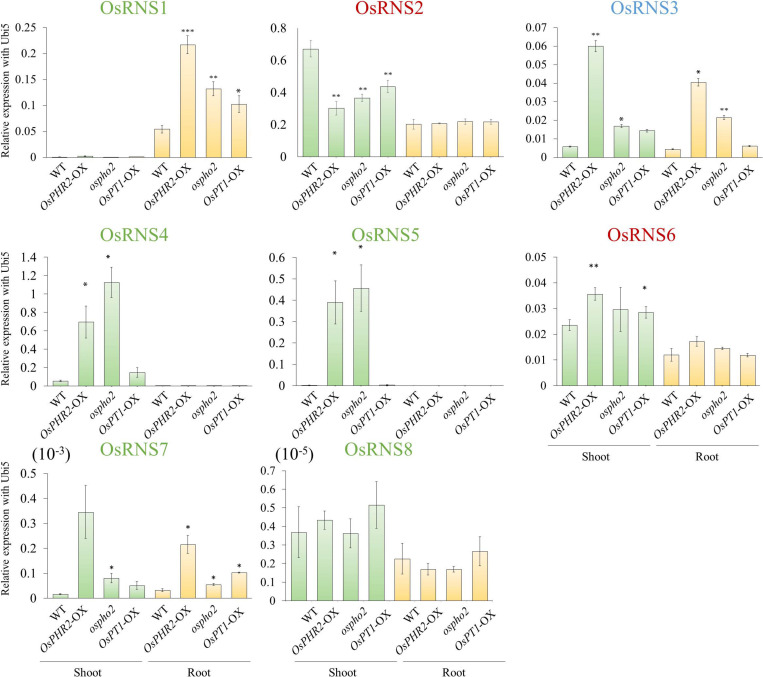
Expression analysis of eight rice *RNS* genes in the phosphate-starvation-induced (PSI) pathway using *OsPHR2*-OX, *ospho2*, and *OsPT1*-OX via qRT-PCR. *X*-axis, PSI mutant and tissues used for qPCR analysis; *Y*-axis, relative expression level compared with *OsUbi5*. Values are means ± SE (*n* = 3), and the asterisk indicates that the values of the mutants or Ox plants differ significantly (*P* < 0.05) compared with their corresponding wild types. ^∗∗^*P* < 0.01, ^∗^*P* < 0.05, based on a *t*-test.

**FIGURE 7:**
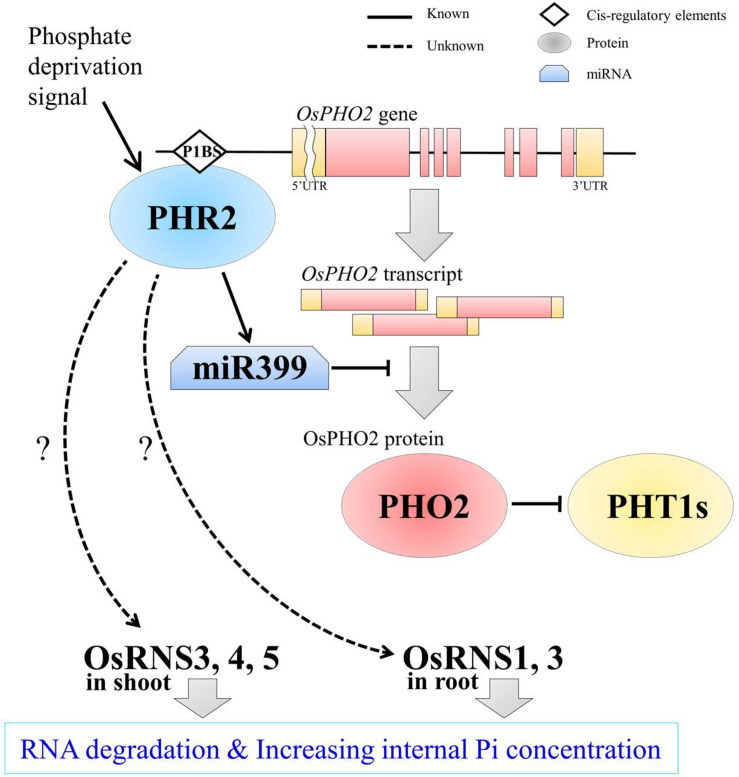
A schematic representation of *OsRNSs* regulation in the PHR2- and PHO2-mediated PSI signaling pathway for increasing internal Pi concentration in rice. PHR2, as a central regulator of the Pi signaling pathway, increases miR399 levels, thereby inhibiting the E2 ligase, *PHO2*, at the post-transcriptional level. Meanwhile, the direct binding of PHR2 on P1BS cis-regulatory element in the promoter region of *PHO2* was reported for the possibility of transcriptional regulation of *PHO2* by PHR2. After the PHO2 protein was generated, it ubiquitinates the phosphate transporters, PHT1s, for maintaining phosphate homeostasis. Our results suggest that *OsRNS1*, *3*, *4*, and *5* are negatively regulated by *OsPHO2* and that the *RNSs* are positively regulated by *OsPHR2* through expression profiling analysis using the mutants. However, it is questionable that Pi contents were enhanced in *OsPHR2* overexpression lines together with the increased amount of the total RNAs including rRNA, the substrate of RNS for rRNA catabolism. The shoot preferential *OsRNS3*, *OsRNS4*, and *OsRNS5* and the root preferential *OsRNS1* and *OsRNS3* seem to be spatially separated and regulated. Finally, it is expected that the accumulation of *OsRNSs* in each tissue will promote RNA degradation and increase the internal Pi concentration. Proteins are represented by circle; cis-regulatory elements, rhombus; mRNA, distorted square; known pathway, solid line; unknown but expected pathway, dotted line.

To determine whether *OsRNS* genes are involved in the PSI signaling pathway via the degradation of RNAs involved in the recycling phosphate sources, we examined their expression patterns in the *rice phosphate starvation response 2* overexpressing line (*OsPHR2*-OX), *rice phosphate 2* (*ospho2)* mutant, and *rice phosphate transporter1* overexpressing line (*OsPT1*-OX) and compared them with those of the wild-type control. To prove the above hypothesis, we acquired an *ospho2* mutant that contains a T-DNA insertion in the 5’UTR of *OsPHO2 from RiceGE*^1^ and *OsPT1*-OX as a high-affinity phosphate transporter from DongA University (Seo et al., 2008; Sun et al., 2012). Moreover, we generated *OsPHR2-OX* transgenic plant and reaffirmed the results that overexpression of *OsPHR2* caused the excessive accumulation of Pi. However, we identified that the RNA concentration increased unlike expectation (Figure 5). The loss of function of *OsPHO2*, a *Tos17* transposon insertion mutant line, led to excessive accumulation of Pi and presented leaf tip necrosis (Zhou et al., 2008; Hu et al., 2011; Cao et al., 2014). *ospho2* mutant by the T-DNA insertion also revealed the excessive accumulation of Pi (Figures 5A,B) and leaf tip necrosis in mature plant (Supplementary Figure S5). Furthermore, RNA concentration was significantly decreased with degraded RNA integrity in the *ospho2* mutant (Figure 5). The function of *OsPT1* was reported in Pi starvation response and *OsPT1-OX* accumulated almost twice as much phosphate (Figure 5). We confirmed the overexpression of *OsPHR2* in *OsPHR2*-OX and of *OsPT1* in *OsPT1*-OX, and the knockout of *OsPHO2* in the *ospho2* mutant by qRT-PCR analysis and morphology (Supplementary Figures S5–S7). Subsequently, we examined the expression patterns of the eight *RNS* genes that exhibited differential expression patterns in PSI signaling gene mutants (Figure 6). We found that the expression of *OsRNS1* alone was significantly altered in the roots of *OsPHR2*-OX, *ospho2*, and *OsPT1*-OX. The expression of *OsRNS4* and *OsRNS5*, which had shoot-preferred expression, was enhanced in the shoots of *OsPHR2*-OX and *ospho2* compared with the wild type, but not in *OsPT1*-OX. *OsRNS3* was upregulated in the tissues of both *OsPHR2*-OX and *ospho2.* Although *OsRNS7* was upregulated in *OsPHR2*-OX and *OsPT1*-OX, its level of expression was very low. There was no significant difference in expression of *OsRNS8* with very low level of expression. *OsRNS2* and *OsRNS6* are class II *RNS* genes with ubiquitous expression. Although the extents were very slight, the latter was positively regulated, while the former was negatively regulated only in the shoots of *OsPHR2*-OX, *ospho2*, and *OsPT1*-OX lines. The induction of the *OsRNS1*, *OsRNS3*, *OsRNS4*, and *OsRNS5* class I *RNS* genes by Pi starvation can be modulated through the *OsPHR2-* and *OsPHO2*-mediated PSI signaling pathway (Figure 6). Overall, we propose that the regulation of the rice *RNS* family of genes might be strongly associated with phosphate recycling and scavenging via RNA degradation under Pi starvation through the *OsPHR2-* and *OsPHO2*-mediated PSI signaling pathway (Figure 7).

## Discussion

*Arabidopsis* comprises four class I *RNS* genes, whereas rice contains six class I *RNS* genes. Among them, with the exception of a class I common *RNS* gene (conserved between rice and *Arabidopsis*), four genes were in the class I monocot-specific *RNS* subgroup and three genes were in the class I dicot-specific *RNS* subgroup. In the class I common *RNS* gene, *AtRNS1* is expressed in most tissues but exhibits stronger expression in seeds and flower, whereas *OsRNS3*, which is the ortholog of *AtRNS1*, is expressed in most tissues, but has a low level of expression in roots and seeds (Bariola et al., 1994). In rice, *OsRNS1* exhibited root-preferred expression, *OsRNS4* had leaf blade-preferred expression, and *OsRNS5*, the tandemly duplicated form of *OsRNS4*, had flower-preferred expression, while *AtRNS4* in the *Arabidopsis* class I dicot-specific *RNS* subgroup showed root-specific expression, and the remaining genes did not show specific expression patterns according to the type of tissues/organs. These results suggest that class I RNS genes in rice might be more precisely regulated according to tissues/organs than those of *Arabidopsis*. Meanwhile, combination of phylogenetic and gene expression analyses suggested that class II RNS genes in rice carry out a housekeeping function in plant species (MacIntosh et al., 2010; Hillwig et al., 2011). *AtRNS2* is a unique *Arabidopsis* class II *RNS* gene that is expressed in all tissues and senescence and Pi starvation at high levels and is localized at an intracellular compartment (Hillwig et al., 2011; Floyd et al., 2017). In rice, the *OsRNS2* and *OsRNS6* class II *RNS* genes are expressed in all tissues and are positively associated with Pi deficiency, similar to *AtRNS2* (Figures 2, 3).

Although previous reports elucidated the functional roles of *RNS* genes in tomato, *Arabidopsis*, and humans associated with senescence, autophagy, tumor suppression, and antifungal activity, respectively (Lers et al., 1998; Acquati et al., 2011; Floyd et al., 2015; Huang et al., 2015; Islam et al., 2019; Singh et al., 2020), the functional roles of *RNS* genes in rice have been poorly discovered. In our study, tissue-preferential regulation in response to Pi deficiency is demonstrated. We found that most of class I monocot-specific *RNS* genes in rice were differentially regulated in response to Pi deficiency according to the type of tissues/organs. *OsRNS1* exhibited root-preferred upregulation under Pi starvation, and *OsRNS4* showed shoot-preferred upregulation under Pi starvation. *AtRNS1* and *AtRNS2* are induced by Pi starvation in shoots and roots, while *AtRNS3*, *AtRNS4*, and *AtRNS5* are not (Taylor et al., 1993; Bariola et al., 1994). It was also reported that the extracellular *RNase LX* and intracellular *RNase Le* from *S. lycopersicum* are induced in tomato suspension cells under Pi starvation (Köck et al., 1998). Furthermore, we found that two class I monocot-specific RNS genes, *OsRNS4* and *OsRNS5*, were upregulated by salt stress and drought stress in addition to responses to Pi deficiency through publicly available transcriptome data analysis (Supplementary Figure S8). It was also reported that *OsRNS4* is involved in abscisic acid (ABA)-mediated responses as well as salinity treatment (Zheng et al., 2014).

Interestingly, the OsRNS4 and OsRNS5 shared high amino acid similarity including the two CAS domains, which are conserved in a “monocot inactive cluster” (MacIntosh et al., 2010; Ramanauskas and Igic, 2017). However, their expression by tissue and response under Pi deficiency had different patterns (Figure 3). It is inferred that tandemly duplicated OsRNS4 and OsRNS5 acquire functional diversity by regulation of promoters with different regulatory elements. Such cases have already been reported. The tandemly duplicated *PRX* genes in rice, *Os1-CysPrxA* and *Os1-CysPrxB*, also have different expression patterns. Although tandemly duplicated *PRX* genes in rice, *Os1-CysPrxA* and *Os1-CysPrxB*, might be functionally redundant due to high amino acid sequence similarity, their anatomical expression patterns are quite different (Gho et al., 2017). It has been reported that their promoters contain unique tissue-preferred CREs: the promoter of *Os1-CysPrxA* has CREs for endosperm preferred expression, while that of *Os1-CysPrxB* has embryo or root-preferred CREs. Thus, OsRNS4 and OsRNS5 are likely to have lost their ability to cleave RNA, and their tight regulation and fine-grained regulatory mechanisms indicate the potential for a role in phosphate deficiency signaling. In addition, our results also suggest that tissue-specific control of the RNS family triggered by gene duplication events in rice exhibits an increase in functional diversity during evolution compared to Arabidopsis.

In addition to the Pi deficiency signal, recent studies have shown that RNA metabolism, including RNA disruption, RNA stability, and RNA processing, plays an important role in responding to abiotic stresses including heat, salt, and nutrient deficiencies (Huang et al., 2015; Kawa and Testerink, 2017; Matsui et al., 2019; Diaz-Baena et al., 2020). Here, we performed various analyses of RNA content, RNase activity, and RNA stability that suggest that RNS induced under Pi deficiency makes Pi acquisition by RNA degradation (Figures 3–5). Our results could also reveal that RNase activity and RNA degradation were enhanced in both Pi-deficient shoots and roots. In addition, it was confirmed that the 25S rRNA fragment was significantly reduced, and the peaks and bands of rRNAs in total RNA were degraded in both the Pi-deficient shoots and roots, resulting in an increase in small RNA fragments (Figures 4D,F). Based on these results, the possibility of providing Pi through rRNA catabolism in *OsRNSs* induced by Pi deficiency was suggested. Meanwhile, several reports have shown that RNS genes play an important role in nitrogen deficiency in various plant species through rRNA catabolism. In nitrogen-deficient wheat, TaRNS2 has been reported to increase RNA degradation and is involved in the catabolism of nucleotides for growth (Melino et al., 2018) and *Rny1*, which is an RNase T2 enzyme from *Saccharomyces cerevisiae*, plays a major role in autophagy-dependent RNA degradation under nitrogen-starvation conditions (Huang et al., 2015). Furthermore, recently, AtRNS1 was reported to be involved in the generation of tRNA-derived fragments (tRFs) under Pi-deficient conditions (Megel et al., 2019). Unfortunately, our data did not analyze the involvement of rice RNS family in the generation of tRFs, but it is necessary to elucidate the association through subsequent studies.

It has been reported that overexpression of *OsPHR2*, loss of function of *OsPHO2*, and overexpression of *OsPT1* have excessively accumulated phosphoric acid in plants (Zhou et al., 2008; Cao et al., 2014). Therefore, it was questioned whether the cause of this excessive accumulation of Pi was affected by *OsRNS*s, so we tried to clarify this through RNA content, Pi content, and RNA degradation analyses between each of the lines and wild type. Although it was confirmed that the Pi content was increased in all three lines, the *ospho2* mutant alone exhibited a significantly decreased RNA concentration and degraded rRNA (Figures 5C,D). Moreover, we confirmed that *OsRNS1*, *3*, *4*, and *5* were strongly enhanced by the *ospho2* mutant and *OsPHR2*-OX, indicating that *OsRNS1*, *3*, *4*, and *5* might be involved in the *OsPHR2*- and *OsPHO2*-mediated PSI signaling pathway. However, it is questionable that Pi contents were enhanced in *OsPHR2* overexpression lines together with increased amount of the total RNAs including rRNA, the substrate of RNS for rRNA catabolism. These results suggest that there are unknown mechanisms for increasing the Pi content or increase of the RNA anabolism in *OsPHR2* overexpression lines.

In summary, we have shown that Pi starvation leads to the induction of *OsRNS* genes encoding ribonucleases and stimulates RNA degradation for Pi acquired in rice by *OsRNS* family genes. In addition, tissue-preferential expression of the *OsRNS* genes under phosphate deficiency plays an important role in RNA remodeling and Pi recycling, and this mechanism is regulated by *OsPHR2* and *OsPHO2*. The role of the *OsRNS* genes in the PSI signaling process has been explained to some extent through the expression analysis of the *OsRNS* gene family in the OsPHR2-OX, OsPT1-OX, and ospho2 mutant lines, but the RNS family affects various stress signals and complex plant homeostasis. Thus, the various features of this family in rice as a model crop plant require further studies.

## Data Availability Statement

The datasets presented in this study can be found in online repositories. The names of the repository/repositories and accession number(s) can be found in the article/Supplementary Material.

## Author Contributions

Y-SG and K-HJ conceived, designed, and supervised the experiments, the data collection, and analyses. Y-SG, HC, and K-HJ performed the experiments, analyzed the data, and wrote the draft of the manuscript. Y-SG and HC performed the molecular analysis. MS, SM, and D-HK provided the reagents, materials, and advice. Y-SG, HC, and K-HJ wrote the final version of the manuscript. All authors contributed to the article and approved the submitted version.

## Conflict of Interest

The authors declare that the research was conducted in the absence of any commercial or financial relationships that could be construed as a potential conflict of interest.

## Footnotes

1 http://signal.salk.edu/cgi-bin/RiceGE

2 https://bioinformatics.psb.ugent.be/plaza/versions/plaza_v4_dicots/

3 http://rice.plantbiology.msu.edu/

4 http://www.ncbi.nlm.nih.gov/geo/

5 http://www.tm4.org/mev/

6 http://www.ncbi.nlm.nih.gov/sra

## Supplementary Material

Supplementary Figure 1 Phylogenetic tree of Class I and Class II RNS family proteins in two monocot and four dicot plants obtained by the neighbor-joining method. The comparison of two monocots (*Rice* and *Z. mays)* and four dicots (*Arabidopsis, B. rapa, Solanum lycopersicum*, and *Medicago truncatula*) RNSs by the phylogenetic tree revealed two classes (i.e., Class I and Class II) of RNS family in plant species and in Class I RNS family, we found three subgroups: Class I dicot specific RNS (purple vertical line), Class I common RNS (blue), and Class I monocot specific RNS (yellow green). Class II RNS was marked as red vertical line. The phylogenetic tree was built using MEGA 7 under the neighbor-joining method.

Supplementary Figure 2 Transcriptome analysis of eight rice *RNS* genes and five *Arabidopsis RNS genes* under phosphate starvation based on public RNA-seq data. Heat map are presented graphically differential expression under log2-(Pi-deficient conditions)/+(Pi-sufficient condition) comparison, or log2-(Pi-resupply condition)/- comparison (left). Red, upregulation of gene expression; green, downregulation of gene expression. Average normalized three FPKM values and three intensity value of *RNS* genes from RNA-seq and microarray data (right). Blue, lowest level of gene expression; yellow, highest level of expression.

Supplementary Figure 3 Expression analysis of the marker gene (*OsPT6*) with root-preferred expression under phosphate station. *X*-axis, tissues and conditions used for qPCR analysis; *Y*-axis, relative expression level compared with *OsUbi5*. Values are means ± SE (*n* = 4) and asterisk indicates that the values of the phosphate starvation differ significantly (*P* < 0.05) compared with phosphate sufficient condition. ^∗∗∗^*P*-value < 0.001, ^∗∗^*P*-value < 0.01, ^∗^*P*-value < 0.05, based on a *t*-test.

Supplementary Figure 4 Analysis of cis-acting regulatory elements in *RNS*-family gene promoters **(A)**, detailed CRE information **(B)**.

Supplementary Figure 1 Schematic position of T-DNA insertional regions on the OsPHO2 genomic structure, expression analysis, and phenotype of the knockout of Phosphate 2 (*pho2*). Schematic position of T-DNA insertional region on the *OsPHO2* genomic structure and genotyping result using a T-DNA primer (TubR3) and gene-specific primers (F/R) in *ospho2* mutant **(A)**. The combination of F and TubR3 primers revealed the T-DNA insertion and that of F and R primers indicates knockout mutation in *OsPHO2* gene. Confirmation for expression level of *OsPHO2* genes in *ospho2* mutant **(B)**. Values are means ± SE (*n* = 3) and asterisk indicates that the *OsPHO2* expression values of *ospho2 mutants* differ significantly (*P* < 0.05) compared with the expression values of WT. *Y*-axis, relative expression level compared to *OsUbi5*. ^∗∗∗^*P*-value < 0.001, ^∗∗^*P*-value < 0.01, ^∗^*P*-value < 0.05, based on a *t*-test. Image of the T5 plants at 10 days after germination being grown in MS media **(C)**. Image of the T5 plants at 90 days after germination being grown in the field condition **(D)**.

Supplementary Figure 6 Expression analysis and phenotype of the overexpression of Phosphate Starvation Response 2 (*OXPHR2*). Confirmation of expression level of *OsPHR2-*OX (A). Values are means ± SE (*n* = 3) and asterisk indicates that the *OsPHR2* expression values of *OsPHR2*-OX differ significantly (*P* < 0.05) compared with the expression values of WT. *Y*-axis, relative expression level compared to *OsUbi5*. ^∗∗∗^*P*-value < 0.001, ^∗∗^*P*-value < 0.01, ^∗^*P*-value < 0.05, based on a *t*-test. Image of the T3 plants at 10 days after germination being grown in MS media **(B)**. Image of the T2 plants at 90 days after germination being grown in the field condition **(C)**.

Supplementary Figure 7 Expression analysis and phenotype of the overexpression of phosphate transporter 1 (*OXPT1*) lines. Confirmation of expression level of *OsPT1-*OX **(A)**. Values are means ± SE (*n* = 3) and asterisk indicates that the *OsPT1* expression values of *OsPT1*-OX differ significantly (*P* < 0.05) compared with the expression values of WT. *Y*-axis, relative expression level compared to *OsUbi5*. ^∗∗∗^*P*-value < 0.001, ^∗∗^*P*-value < 0.01, ^∗^*P*-value < 0.05, based on a *t*-test. Image of the T6 plants at 10 days after germination being grown in MS media **(B)**. Image of the T6 plants at 90 days after germination being grown in the field condition **(C)**.

Supplementary Figure 8 Meta-expression analysis in response to various abiotic stresses based on Affymetrix array platforms. Red, upregulation of gene expression; green, downregulation of gene expression.

Supplementary Table 1 Summary of the primer sequences used for qPCR analyses in Figures 2, 3, 6 and Supplementary Figures S2, S5.

Supplementary Table 2 Detailed information of the *RNS* genes in rice and *Arabidopsis*.
