# Supplementary figures and images for: Phosphate-Starvation-Inducible S-Like RNase Genes in Rice Are Involved in Phosphate Source Recycling by RNA Decay
Source: Front Plant Sci. 2020 Nov 30;11:585561. doi: 10.3389/fpls.2020.585561 (PMC7793952; doi:10.3389/fpls.2020.585561)

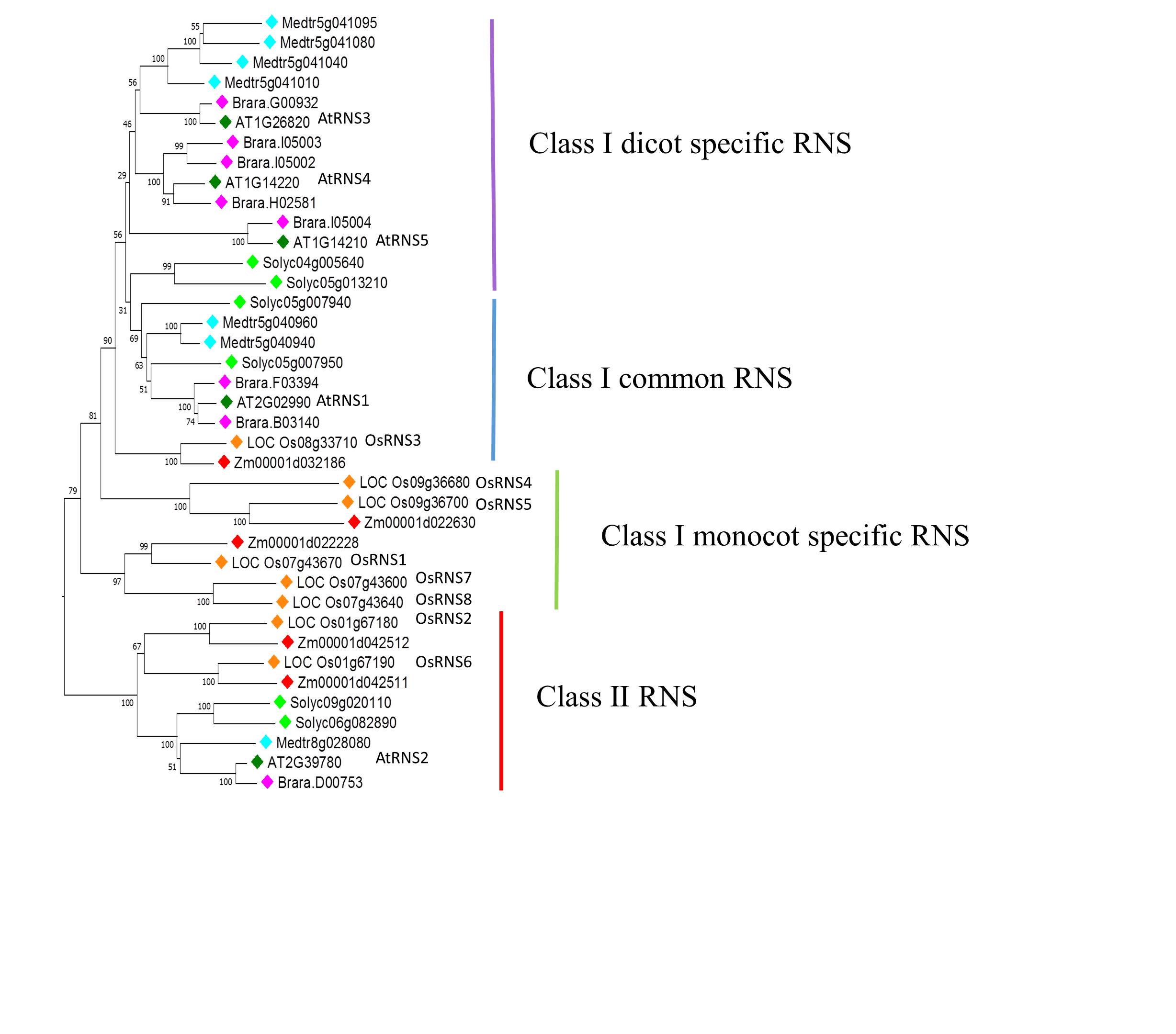

Supplement: Supplementary Figure 1 — Phylogenetic tree of Class I and Class II RNS family proteins in two monocot and four dicot plants obtained by the neighbor-joining method. The comparison of two monocots (Rice and Z. mays) and four dicots (Arabidopsis, B. rapa, Solanum lycopersicum, and Medicago truncatula) RNSs by the phylogenetic tree revealed two classes (i.e., Class I and Class II) of RNS family in plant species and in Class I RNS family, we found three subgroups: Class I dicot specific RNS (purple vertical line), Class I common RNS (blue), and Class I monocot specific RNS (yellow green). Class II RNS was marked as red vertical line. The phylogenetic tree was built using MEGA 7 under the neighbor-joining method. [file Image_1.JPEG]

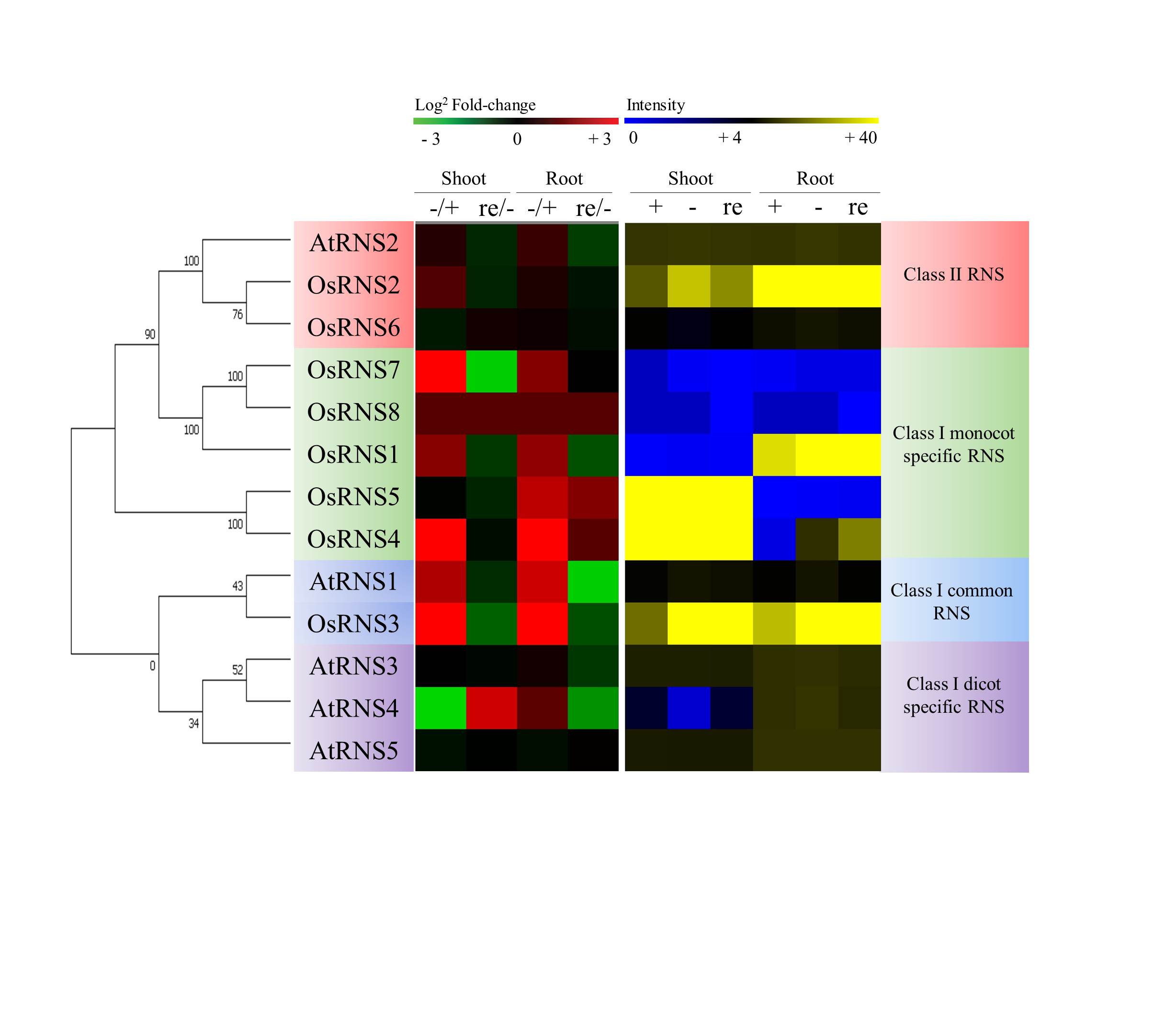

Supplement: Supplementary Figure 2 — Transcriptome analysis of eight rice RNS genes and five Arabidopsis RNS genes under phosphate starvation based on public RNA-seq data. Heat map are presented graphically differential expression under log2-(Pi-deficient conditions)/+(Pi-sufficient condition) comparison, or log2-(Pi-resupply condition)/- comparison (left). Red, upregulation of gene expression; green, downregulation of gene expression. Average normalized three FPKM values and three intensity value of RNS genes from RNA-seq and microarray data (right). Blue, lowest level of gene expression; yellow, highest level of expression. [file Image_2.JPEG]

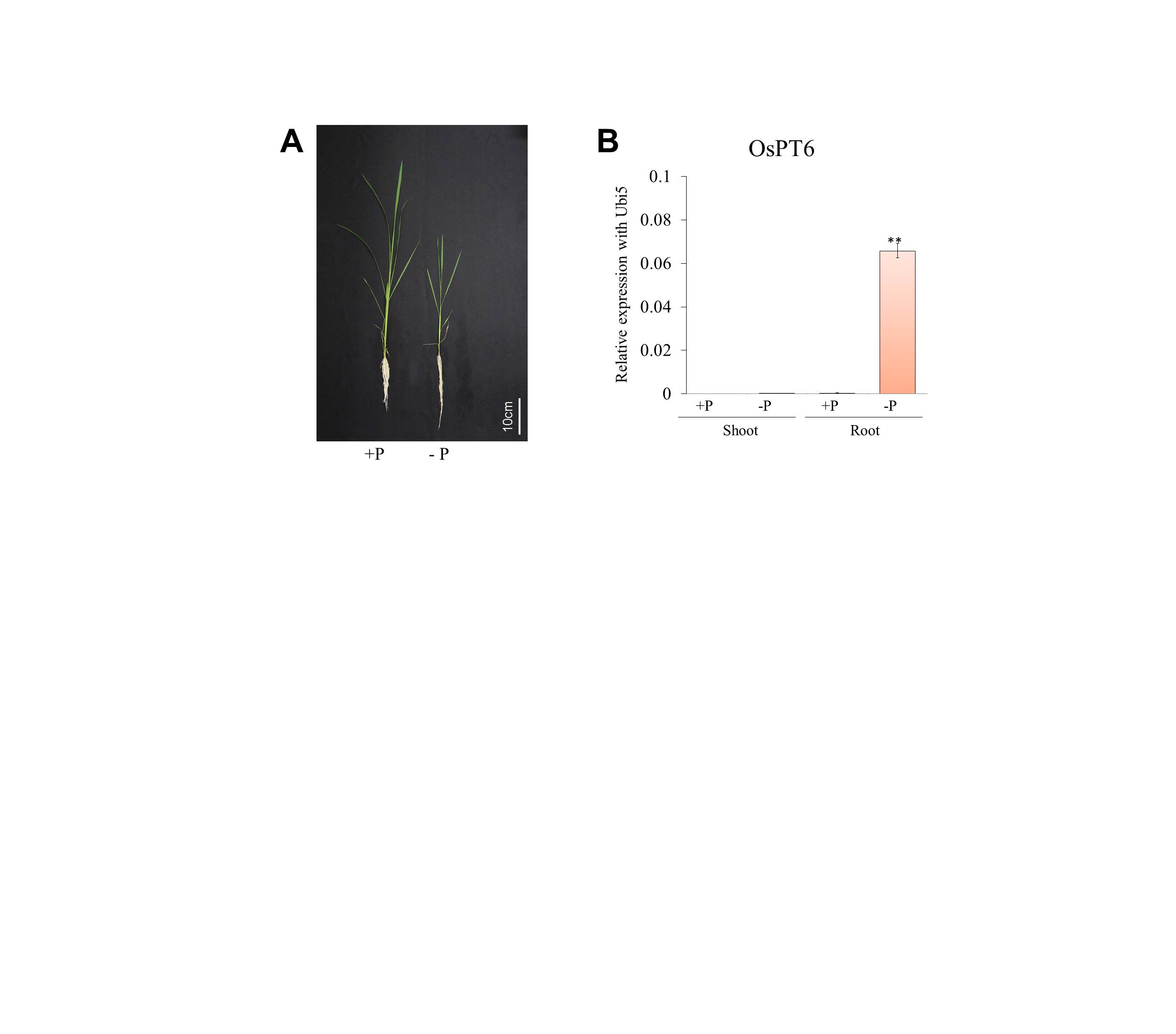

Supplement: Supplementary Figure 3 — Expression analysis of the marker gene (OsPT6) with root-preferred expression under phosphate station. X-axis, tissues and conditions used for qPCR analysis; Y-axis, relative expression level compared with OsUbi5. Values are means ± SE (n = 4) and asterisk indicates that the values of the phosphate starvation differ significantly (P < 0.05) compared with phosphate sufficient condition. ∗∗∗P-value < 0.001, ∗∗P-value < 0.01, ∗P-value < 0.05, based on a t-test. [file Image_3.JPEG]

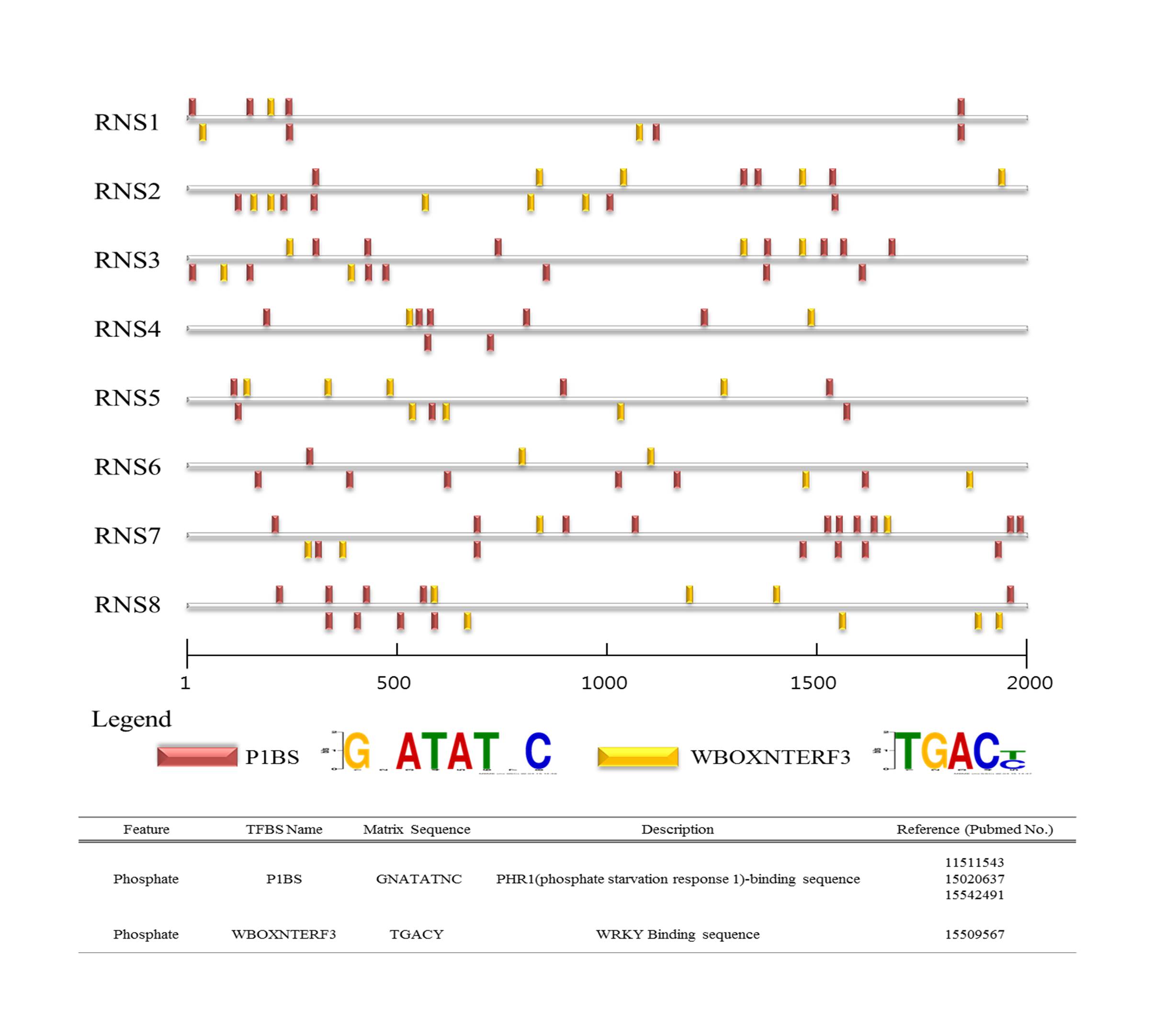

Supplement: Supplementary Figure 4 — Analysis of cis-acting regulatory elements in RNS-family gene promoters (A), detailed CRE information (B). [file Image_4.JPEG]

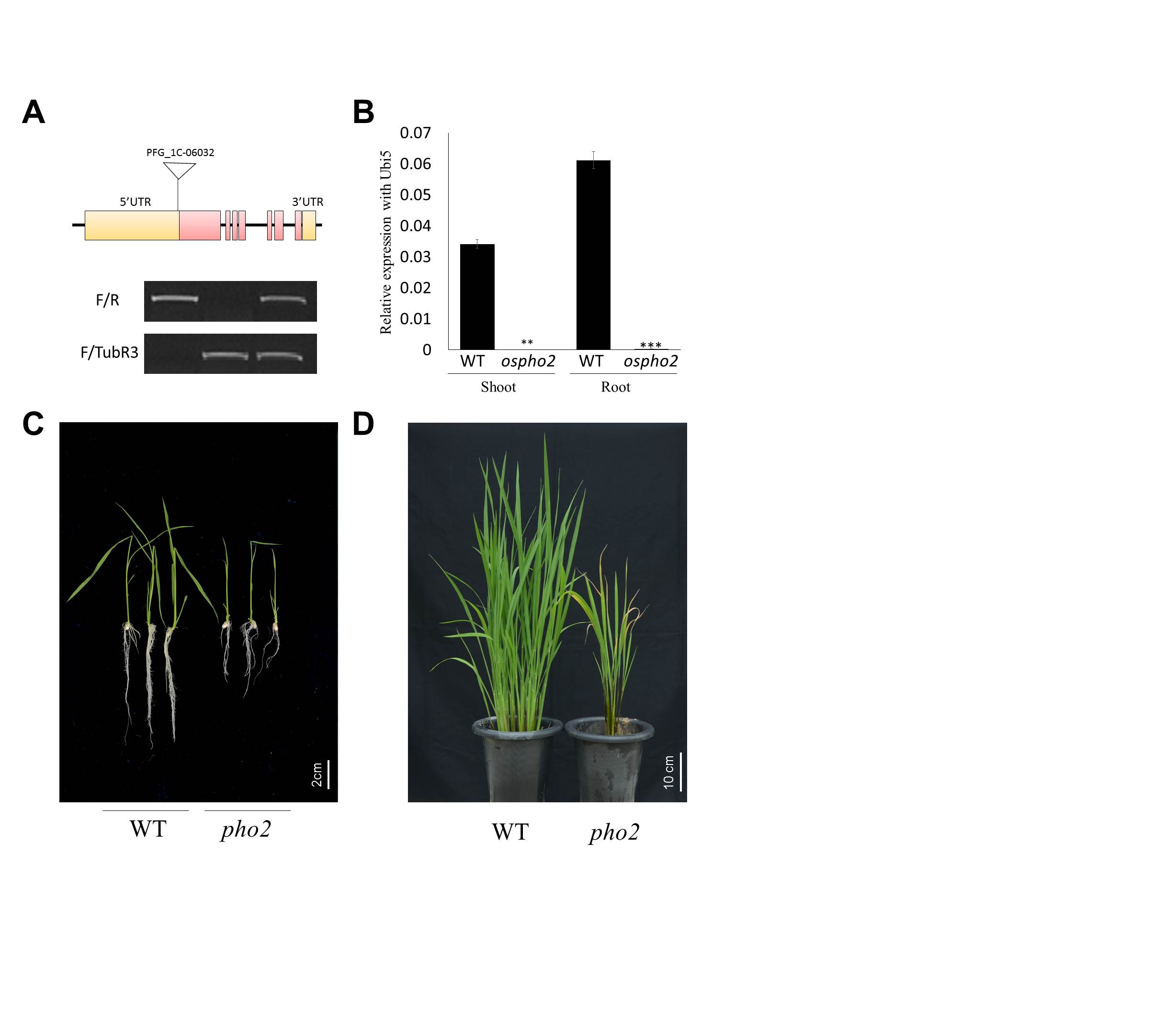

Supplement: Supplementary Figure 1 — Schematic position of T-DNA insertional regions on the OsPHO2 genomic structure, expression analysis, and phenotype of the knockout of Phosphate 2 (pho2). Schematic position of T-DNA insertional region on the OsPHO2 genomic structure and genotyping result using a T-DNA primer (TubR3) and gene-specific primers (F/R) in ospho2 mutant (A). The combination of F and TubR3 primers revealed the T-DNA insertion and that of F and R primers indicates knockout mutation in OsPHO2 gene. Confirmation for expression level of OsPHO2 genes in ospho2 mutant (B). Values are means ± SE (n = 3) and asterisk indicates that the OsPHO2 expression values of ospho2 mutants differ significantly (P < 0.05) compared with the expression values of WT. Y-axis, relative expression level compared to OsUbi5. ∗∗∗P-value < 0.001, ∗∗P-value < 0.01, ∗P-value < 0.05, based on a t-test. Image of the T5 plants at 10 days after germination being grown in MS media (C). Image of the T5 plants at 90 days after germination being grown in the field condition (D). [file Image_5.JPEG]

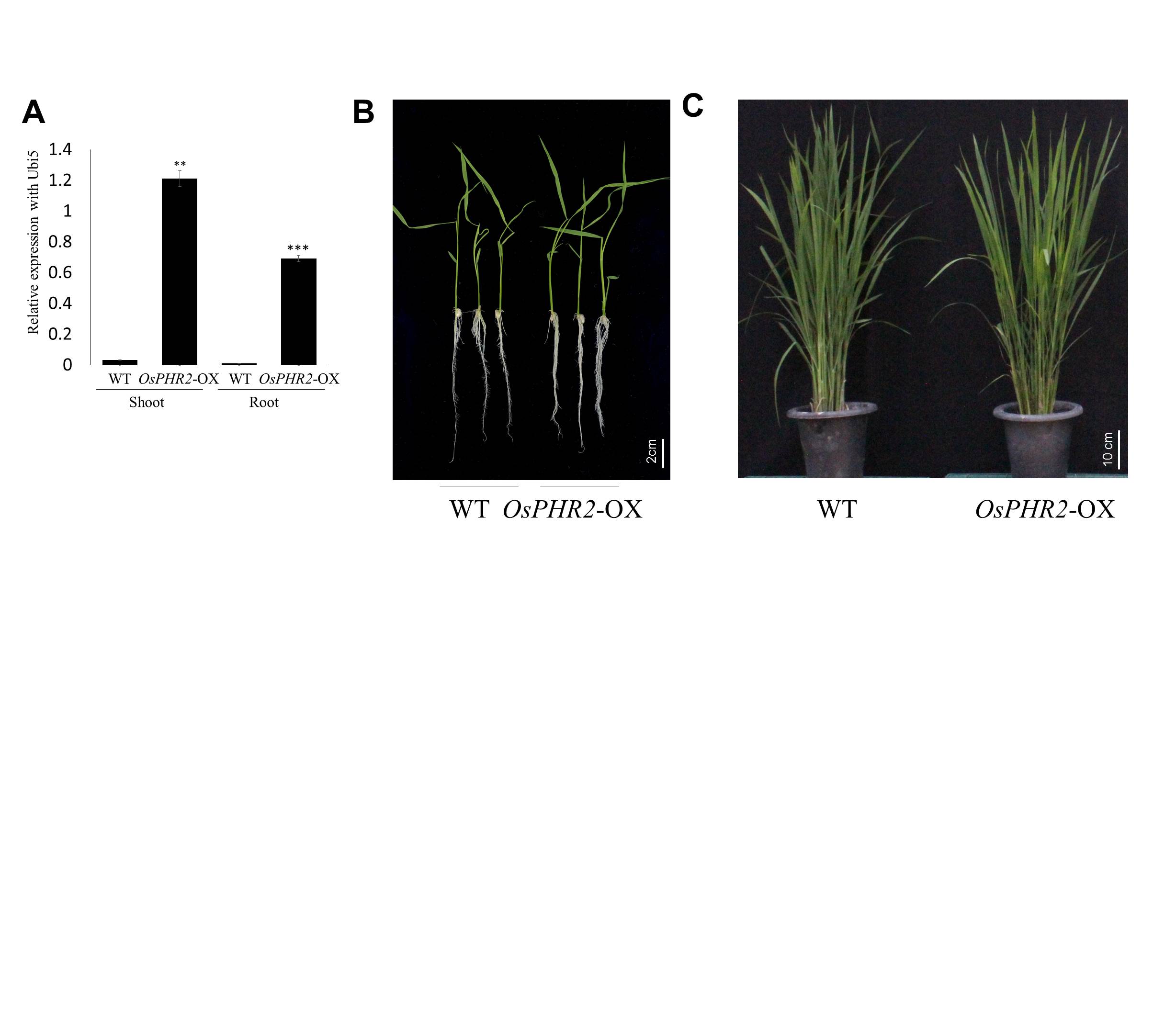

Supplement: Supplementary Figure 6 — Expression analysis and phenotype of the overexpression of Phosphate Starvation Response 2 (OXPHR2). Confirmation of expression level of OsPHR2-OX (A). Values are means ± SE (n = 3) and asterisk indicates that the OsPHR2 expression values of OsPHR2-OX differ significantly (P < 0.05) compared with the expression values of WT. Y-axis, relative expression level compared to OsUbi5. ∗∗∗P-value < 0.001, ∗∗P-value < 0.01, ∗P-value < 0.05, based on a t-test. Image of the T3 plants at 10 days after germination being grown in MS media (B). Image of the T2 plants at 90 days after germination being grown in the field condition (C). [file Image_6.JPEG]

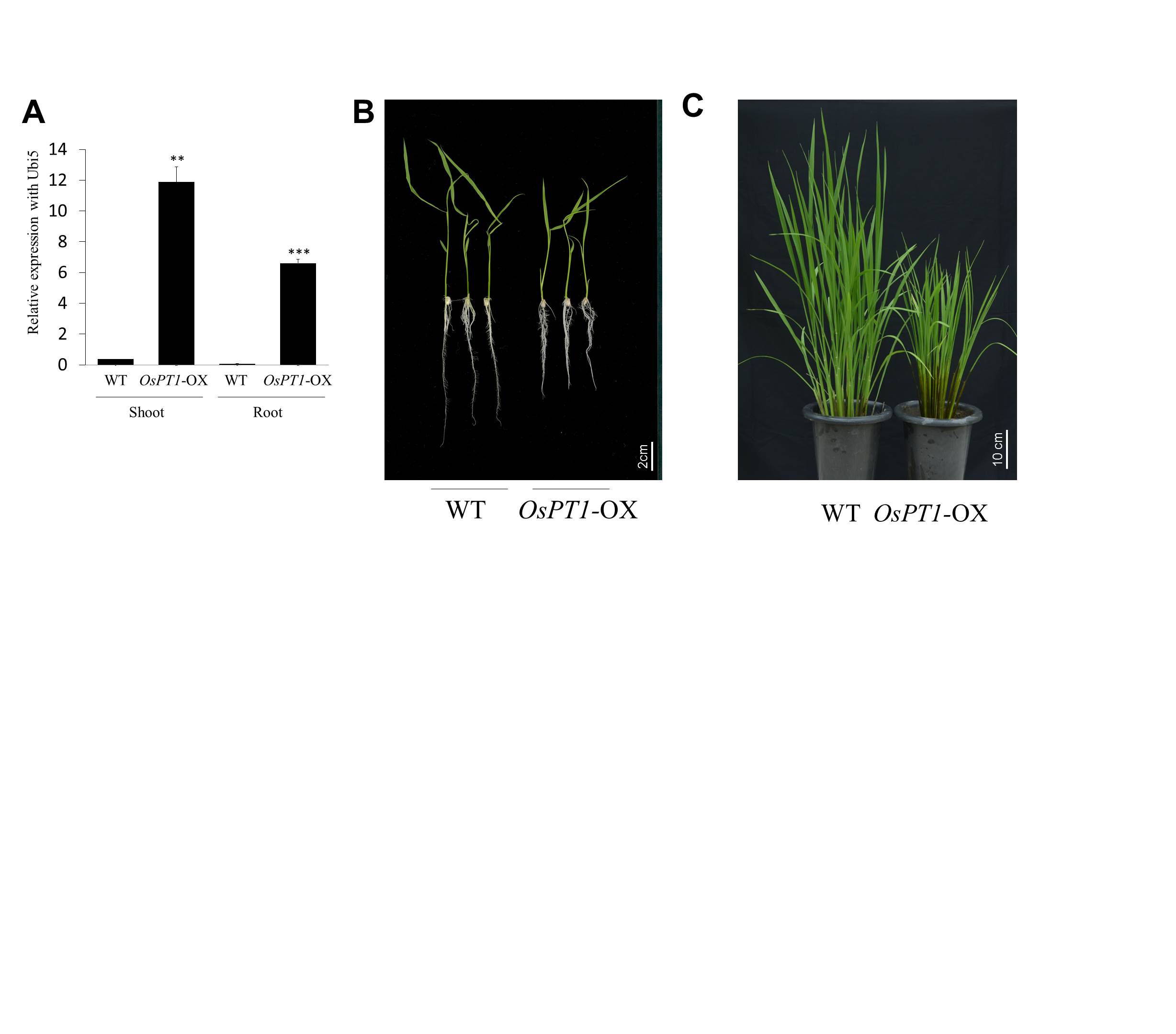

Supplement: Supplementary Figure 7 — Expression analysis and phenotype of the overexpression of phosphate transporter 1 (OXPT1) lines. Confirmation of expression level of OsPT1-OX (A). Values are means ± SE (n = 3) and asterisk indicates that the OsPT1 expression values of OsPT1-OX differ significantly (P < 0.05) compared with the expression values of WT. Y-axis, relative expression level compared to OsUbi5. ∗∗∗P-value < 0.001, ∗∗P-value < 0.01, ∗P-value < 0.05, based on a t-test. Image of the T6 plants at 10 days after germination being grown in MS media (B). Image of the T6 plants at 90 days after germination being grown in the field condition (C). [file Image_7.JPEG]

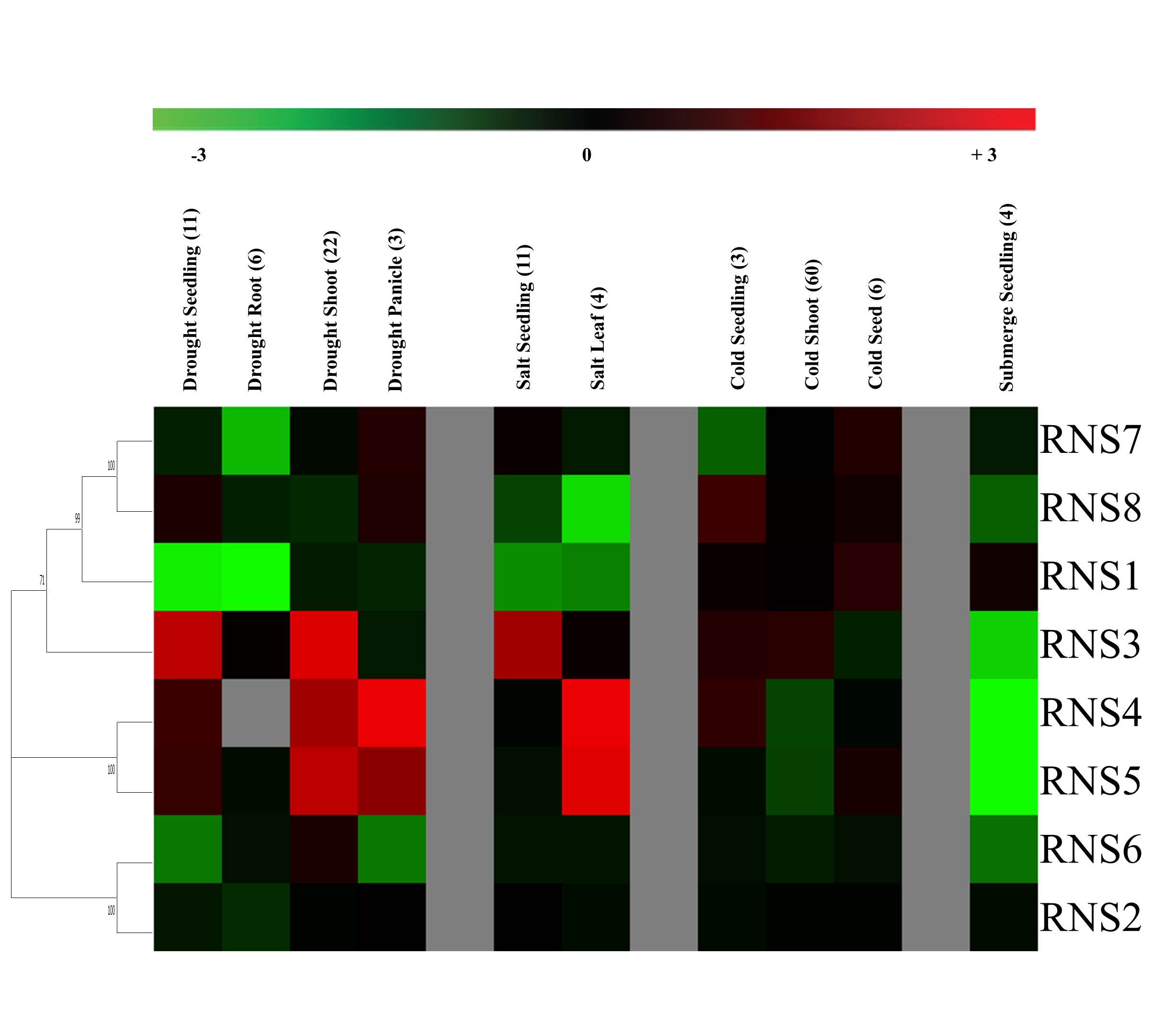

Supplement: Supplementary Figure 8 — Meta-expression analysis in response to various abiotic stresses based on Affymetrix array platforms. Red, upregulation of gene expression; green, downregulation of gene expression. [file Image_8.JPEG]
