# Supplementary material for: Phosphate-Starvation-Inducible S-Like RNase Genes in Rice Are Involved in Phosphate Source Recycling by RNA Decay
Source: Front Plant Sci. 2020 Nov 30;11:585561. doi: 10.3389/fpls.2020.585561 (PMC7793952; doi:10.3389/fpls.2020.585561)
Supplement: Supplementary Table 1 — Summary of the primer sequences used for qPCR analyses in Figures 2, 3, 6 and Supplementary Figures S2, S5. [file Table_1.DOCX]

Table.S1 Summary of primer sequences used for qPCR analyses in Figures 2, 3, 6, S2-S6.

| Purpose | Gene symbol | LOC_ID | Forward primer(5'-3') | Reverse primer(5'-3') |
| --- | --- | --- | --- | --- |
| RT-PCR | OsRNS1 | LOC_Os07g43670 | GAC GAC GAG AGC TAC TCC CT | CAC CTG GAA CAG CTG CGT CT |
| RT-PCR | OsRNS2 | LOC_Os01g67180 | TGC TGC GCC ATC AAC GGA TG | TTC TCA AGC GTC GGC TTC AG |
| RT-PCR | OsRNS3 | LOC_Os08g33710 | AGG ACT ACG ACT TCT TCT TC | AGG GGT CGA ACT CGC TGT CA |
| RT-PCR | OsRNS4 | LOC_Os09g36680 | TGA CTC GTC AGA GAA CAC AG | ACA CTT GAT GTT GCT CCA GT |
| RT-PCR | OsRNS5 | LOC_Os09g36700 | GTA CAG CGT GAA GAA GAT CA | TCT TGA AAG GGT GGA AGA GGA |
| RT-PCR | OsRNS6 | LOC_Os01g67190 | AAG ACG ATA CTC GAG GAG TA | GCG AGG ATC TTG GTA AGG TT |
| RT-PCR | OsRNS7 | LOC_Os07g43600 | GCA GAT CCA GGA CCT GGT GA | GCG AGG ATC TTG GTA AGG TT |
| RT-PCR | OsRNS8 | LOC_Os07g43640 | AAG CAG CTC GAC CAG AAA TG | AAC GTG TAG GTC TTC ACG TC |
| RT-PCR | OsUbi5 | LOC_Os01g22490 | GCA CAA GCA CAA GAA GGT GA | GCC TGC TGG TTG TAG ACG TA |
| RT-PCR | OsSQD2 | LOC_Os01g04920 | CCT TCT TCT GGA TTC CTC TC | AGT TCA CCA CAA AAG ATA CAG TAG |
| RT-PCR | OsPT6 | LOC_Os08g45000 | GCC CCT GCA AAC TGT ACT G | AGC CAG GCC AGT TAT ATA TCA AC |
| Over-expression transgenic plant | OsPHR2 | LOC_Os07g25710 | GGATCCACTAGTGTTAGAGGCCATTCTCTGTAACAC | CGAGCTGCCTCCGTTAGGAGGGATTGCAGCAGC |
| RT-PCR | OsPHR2 | LOC_Os07g25710 | CGC TTT GTA GAT GCT GTC AAT C | GAT CTA TTG ATG GTA TGT CCT C |
| Genotyping | OsPHO2 | LOC_Os05g48400 | ACA CAA CAG TGA CCA GGT CA | GGA AGT CCA GAA CGA TTC AA |
| Genotyping | TubR3 |  | - | GGT GAA TGG CAT CGT TTG AA |
| RT-PCR | OsPHO2 | LOC_Os05g48400 | CGA GGA GCA CAC TAC TGA TG | AAT GTT GAA TCA TGA GCA CC |
| RT-PCR | OsPT1 | LOC_Os03g05620 | GCG AAC TTC GGG CCA AAC AG | GTC ATG ATC GTC CCG AGG AA |
