# Supplementary material for: Phosphate-Starvation-Inducible S-Like RNase Genes in Rice Are Involved in Phosphate Source Recycling by RNA Decay
Source: Front Plant Sci. 2020 Nov 30;11:585561. doi: 10.3389/fpls.2020.585561 (PMC7793952; doi:10.3389/fpls.2020.585561)
Supplement: Supplementary Table 2 — Detailed information of the RNS genes in rice and Arabidopsis. [file Table_2.DOCX]

Table.S2 Detailed information of RNS gene in rice and Arabidopsis

| Class | Gene symbol | MSU_ID (Os) | RAP_ID (Os) | Gene symbol (At) | Tair_ID (At) |
| --- | --- | --- | --- | --- | --- |
| Class I | OsRNS1 | LOC_Os07g43670 | Os07g0630400 |  |  |
| Class I | OsRNS3 | LOC_Os08g33710 | Os08g0434100 | AtRNS1 | AT2G02990 |
|  |  |  |  | AtRNS3 | AT1G26820 |
|  |  |  |  | AtRNS4 | AT1G14220 |
|  |  |  |  | AtRNS5 | AT1G14210 |
| Class I | OsRNS4 | LOC_Os09g36680 | Os09g0537700 |  |  |
| Class I | OsRNS5 | LOC_Os09g36700 | Os09g0538000 |  |  |
| Class I | OsRNS7 | LOC_Os07g43600 | Os07g0629300 |  |  |
| Class I | OsRNS8 | LOC_Os07g43640 | Os07g0629900 |  |  |
| Class II | OsRNS2 | LOC_Os01g67180 | Os01g0897200 | AtRNS2 | AT2G39780 |
| Class II | OsRNS6 | LOC_Os01g67190 | Os01g0897300 |  |  |
